# The signal transducer CD24 suppresses the germ cell program and promotes an ectodermal rather than mesodermal cell fate in embryonal carcinomas

**DOI:** 10.1002/1878-0261.13066

**Published:** 2021-08-02

**Authors:** Margaretha A. Skowron, Teresa K. Becker, Lukas Kurz, Sina Jostes, Felix Bremmer, Florian Fronhoffs, Kai Funke, Gamal A. Wakileh, Melanie R. Müller, Aaron Burmeister, Thomas Lenz, Anja Stefanski, Kai Stühler, Patrick Petzsch, Karl Köhrer, Peter Altevogt, Peter Albers, Glen Kristiansen, Hubert Schorle, Daniel Nettersheim

**Affiliations:** ^1^ Department of Urology Urological Research Laboratory Translational UroOncology Medical Faculty and University Hospital Düsseldorf Heinrich Heine University Düsseldorf Germany; ^2^ Department of Oncological Science Icahn School of Medicine at Mount Sinai Hess Center for Science and Medicine New York NY USA; ^3^ Institute of Pathology University Medical Center Goettingen Germany; ^4^ Institute of Pathology University Hospital Bonn Germany; ^5^ Department of Developmental Pathology Institute of Pathology University Hospital Bonn Germany; ^6^ Department of Urology University Hospital Ulm Germany; ^7^ Molecular Proteomics Laboratory Heinrich‐Heine‐University Düsseldorf Germany; ^8^ Genomics & Transcriptomics Lab Heinrich Heine University Düsseldorf Germany; ^9^ Skin Cancer Unit German Cancer Research Center (DKFZ) Heidelberg Germany; ^10^ Department of Dermatology, Venereology and Allergology University Medical Center Mannheim Ruprecht‐Karl University Heidelberg Germany; ^11^ Department of Urology Medical Faculty and University Hospital Düsseldorf, Heinrich Heine University Düsseldorf Germany

**Keywords:** CD24, differentiation, embryonal carcinoma, epigenetics, germ cell tumors, microenvironment

## Abstract

Testicular germ cell tumors (GCTs) are stratified into seminomas and nonseminomas. Seminomas share many histological and molecular features with primordial germ cells, whereas the nonseminoma stem cell population—embryonal carcinoma (EC)—is pluripotent and thus able to differentiate into cells of all three germ layers (teratomas). Furthermore, ECs are capable of differentiating into extra‐embryonic lineages (yolk sac tumors, choriocarcinomas). In this study, we deciphered the molecular and (epi)genetic mechanisms regulating expression of *CD24*, a highly glycosylated signaling molecule upregulated in many cancers. *CD24* is overexpressed in ECs compared with other GCT entities and can be associated with an undifferentiated pluripotent cell fate. We demonstrate that *CD24* can be transactivated by the pluripotency factor SOX2, which binds in proximity to the CD24 promoter. In GCTs, *CD24* expression is controlled by epigenetic mechanisms, that is, histone acetylation, since *CD24* can be induced by the application histone deacetylase inhibitors. Vice versa, *CD24* expression is downregulated upon inhibition of histone methyltransferases, E3 ubiquitin ligases, or bromodomain (BRD) proteins. Additionally, three‐dimensional (3D) co‐cultivation of EC cells with microenvironmental cells, such as fibroblasts, and endothelial or immune cells, reduced *CD24* expression, suggesting that crosstalk with the somatic microenvironment influences *CD24* expression. In a CRISPR/Cas9 deficiency model, we demonstrate that CD24 fulfills a bivalent role in differentiation via regulation of homeobox, and phospho‐ and glycoproteins; that is, it is involved in suppressing the germ cell/spermatogenesis program and mesodermal/endodermal differentiation, while poising the cells for ectodermal differentiation. Finally, blocking CD24 by a monoclonal antibody enhanced sensitivity toward cisplatin in EC cells, including cisplatin‐resistant subclones, highlighting CD24 as a putative target in combination with cisplatin.

## Graphical abstract

This study presents the (epi)genetic mechanisms regulating the expression of the signaling transducer *CD24* in embryonal carcinoma (EC), a germ cell tumor subtype with embryonic stem cell‐like features. We demonstrated that *CD24* expression can be transactivated by the pluripotency factor SOX2 and is involved in suppressing the germ cell program during formation of ECs, while promoting ectodermal differentiation.

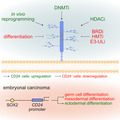

**Abbreviations**
3D: three-dimensional
5mC: 5-methylcytosine
BRD: bromodomain-containing protein
CC: choriocarcinoma
CD24: cluster of differentiation 24
ChiP: chromatin immunoprecipitation
CpG: 5'-cytosine-phosphate-guanine-3'
E3-UL: E3 ubiquitin ligase
EC: embryonal carcinoma
EMT: epithelial-to-mesenchymal transition
FBS: fetal bovine serum
FC: fold change
FDR: false discovery rate
GCNIS: germ cell neoplasia in situ
GCT: germ cell tumor
GEO: gene expression omnibus
GO: gene ontology
GPI: glycosylphosphatidylinositol
HDAC: histone deacetylase
HDM: histone demethylase
HMT: histone methyltransferase
IC_50_
: half-maximal inhibitory concentration
IF: immunofluorescent staining
IHC: immunohistochemistry
KEGG: Kyoto encyclopedia of genes and genomes
LC-MS: liquid chromatography–mass spectrometry
LFQ: label-free quantitation
MS-PCR: methylation-specific polymerase chain reaction
NTT: normal testis tissues
PBS: phosphate-buffered saline
PCA: principal component analysis
PEI: polyethylenimine
PI: propidium iodide
qRT-PCR: quantitative real-time polymerase chain reaction
RA: all-trans retinoic acid
RSEM: RNA-seq by expectation–maximization
RT: room temperature
SAM: significance analysis of microarrays
SD: standard deviation
STR: short tandem repeat
TCGA: The Cancer Genome Atlas
TE: teratoma
XTT: 2,3-bis(2-methoxy-4-nitro-5-sulfophenyl)-5-[(phenylamino)carbonyl]-2H-tetrazolium
YST: yolk sac tumor
Δ: delta; here: *CD24* deficiency

## 1 Introduction

Type II germ cell tumors (GCTs) are the most common neoplasia in young men of age 14–45 years. It is generally accepted that GCTs arise from a common precursor lesion, called germ cell neoplasia *in situ* (GCNIS), eventually developing into seminomas or non‐seminomas [1]. The non‐seminomatous stem cell‐like embryonal carcinomas (ECs) can further differentiate into teratomas (TE), yolk‐sac tumors (YSTs), or choriocarcinomas (CC) [2, 3]. Orchiectomy followed by chemo‐ or radiotherapy is a widely used procedure in the treatment of type II GCTs, leading to high cure rates of up to 90% [3]. Nevertheless, about 10–15% of patients with progressive disease relapse as a result of drug resistance and are condemned for a poor prognosis and a short survival of only a few months [4].

Cluster of differentiation 24 (CD24) is a small, mucin‐like glycosylphosphatidylinositol (GPI)‐anchored membrane molecule that is discussed to function in both signal transduction and adhesion. This glycoprotein is mainly expressed on the surface of hematopoietic, neural, muscular, and epithelial cells [5]. Moreover, CD24 has been implicated in tumor metastasis, as fucosylated CD24 interacts with P‐ and E‐selectin, allowing invasion of tumor cells to distal sites [6]. High expression or amplifications of *CD24* have been described in a variety of solid malignancies, such as non‐small‐cell lung carcinoma, gliomas, breast cancer, retinoblastoma, hepatocellular carcinoma, renal cell carcinoma, cervical carcinoma, prostate cancer, urothelial carcinoma, pineal parenchymal tumors, and ovarian cancer [7, 8, 9, 10, 11, 12, 13, 14, 15, 16, 17, 18]. An exception is multiple myeloma, where CD24 was rather downregulated compared with normal B‐cell cell lines [19, 20]. This can be explained by the fact that *CD24* is expressed in pre‐B‐lymphocytes, remains expressed in mature resting B cells, and becomes downregulated during the maturation process to plasma cells, which are terminally differentiated B cells [21].

Previously, we could identify high *CD24* expression in the EC cell lines NCCIT, 2102EP, and GCT27, but only weak expression in JAR cells (CC) [22]. Similar observations were found in a cohort of 24 GCT patient samples, where *CD24* was predominantly upregulated in non‐seminoma compared with seminoma [23]. However, to our knowledge, no study has investigated the molecular role of CD24 in GCTs. Hence, in this study, we investigated the putative function of CD24 and its interaction partners in (cisplatin‐resistant) GCT cell lines and further deciphered the molecular consequences of a CRISPR/Cas9‐mediated *CD24* deficiency in EC cell lines.

## 2 Materials and methods

The study methodologies conformed to the standards set by the Declaration of Helsinki and were approved by the local ethics committees.

### 2.1 Cell culture

Germ cell tumors and non‐cancerous cell lines were received from and were cultivated as described in Table S1 and as published [24]. Short tandem repeat (STR) profiles of all cell lines are checked on a regular basis and are available upon reasonable request. All cell lines are checked for mycoplasma contamination on a regular basis by a PCR strategy.

### 2.2 Three‐dimensional cell co‐cultivation and cell sorting

A detailed description has been published previously [25]. Briefly, a total amount of 3 × 10^3^ cells per 40‐μL drop have been plated onto an inverted lid of a 15‐cm cell culture plate. Hanging drops were incubated at 37 °C in a 5% CO_2_ incubator for 72 h. For cell sorting, an initial ratio of GCT cell lines to microenvironmental cells of 30 : 70 was used. Cell sorting into GFP^+^ (GCTs) and mCherry^+^/DeepRed^+^ (microenvironmental) cells was performed on a MoFlo XDP (Beckman Coulter, Krefeld, Germany).

### 2.3 Drug applications

See Table S1 for an overview of drugs used in this study, their solvents, concentrations, and application schemes (Table S1). Individual IC_50_ concentrations were determined by XTT assays in quadruplicates and calculated by ‘graphpad prism 8.0’ as described previously [26, 27].

### 2.4 Generation of GFP‐ and mCherry‐expressing GCT and microenvironmental cells

One day prior transfection, 293T cells were seeded into 10 cm plates. For the transfection, 6 µg pczVSV‐G plasmid [28], 6 µg of pCD/NL‐BH∆1 plasmid (Addgene #41791 [29]), and 6 µg of puc2CL6EGIP [30] or pLV‐mCherry plasmid (Addgene #36084) were incubated in supplement‐free DMEM. In parallel, a 45 µg·µL^−1^ of polyethylenimine (PEI; Sigma‐Aldrich, Taufkirchen, Germany) was added to supplement‐free DMEM. Both mixes were combined and incubated for 20 min at room temperature (RT) before being added to 293T cells. The next day, 1 × 10^5^ GCT (for GFP transduction) or microenvironmental cells (for mCherry transduction) were seeded onto 6 wells. 48 h post‐transfection, virus‐containing supernatant was strained through a 0.45‐µm filter and 500 µL of virus per well was added to each well and incubated for 24 h. After exchanging to standard cell culture medium, cells were further incubated for 48 h. GFP‐positive GCT cells were selected by puromycin (1 µg·mL^−1^; Sigma‐Aldrich) for 10–14 days. Additionally, the GFP+ (TCam‐2, 2102EP, NCCIT, JAR, JEG3, 1411H, GCT72) or mCherry+ (HUVEC, JURKAT, THP‐1) cell populations were sorted by flow cytometry to increase purity. Primary fibroblast cultures (HVHF2) were stained by ‘CellTracker CM‐Dil Dye’ for microscopy and by ‘CellTracker DeepRed Dye’ for cell sorting according to the manufacturer’s protocol (both from Thermo Fisher Scientific, Schwerte, Germany).

### 2.5 Polarization of M2 macrophages

THP‐1 cells (Sigma‐Aldrich) were differentiated into macrophages as described by Genin *et al*. [31] by 24 h incubation with 150 nm phorbol 12‐myristate 13‐acetate (PMA; Sigma‐Aldrich) in RPMI medium. Polarization into M2 macrophages was acquired by incubation with 20 ng·mL^−1^ of IL‐4 and 20 ng·mL^−1^ of IL‐13 (both from R&D Systems, Wiesbaden, Germany) for 72 h.

### 2.6 XTT viability assay

XTT viability assays were performed as described previously [32]. Briefly, 3 × 10^3^ cells were used for each replicate. Drugs were added once 24 h before first XTT measurement. Viability was measured over 96 h in quadruplicates.

### 2.7 Transwell migration assay

Before harvesting the cells, culture media were exchanged the day before to ‘starvation medium’ containing only 2% FBS and 1% penicillin/streptomycin. Cells were incubated overnight at 37 °C. To measure the migratory capacity, transwell culture inserts (8 µm pore size ‘ThinCerts’, Greiner Bio‐One, Frickenhausen, Germany) were used in 24‐well plates (Greiner Bio‐One). In each insert, 2.5 × 10^4^ cells were seeded in triplicates in 200 µL ‘starvation medium’. 600 µL standard culture medium was added to the lower chamber. After 24 h, cells were fixed in ice‐cold 100% methanol for 10 min at RT and stained with crystal violet (1 g/100 mL) at RT for 15 min. Inserts were washed twice with PBS, and the inside of the inserts was wiped with cotton swabs. Fixed cells on the outside of the membrane were lysed in 200 µL 10% acetic acid for 10 min at RT, of which 150 µL was transferred to a 96‐well plate and the absorbance was measured at 595 nm (iMark Microplate Absorbance Reader, Bio‐Rad, Feldkirchen, Germany).

### 2.8 Adhesion assay

24‐well plates (Greiner Bio‐One) were blocked with 1.5% bovine serum albumin in PBS for 1 h at RT. Hereafter, supernatants were removed and cells (2 × 10^5^) were seeded in triplicates and incubated for 1 h at 37 °C. Non‐adherent cells were removed by aspiration, and wells were washed twice with PBS. Remaining cells were fixed in ice‐cold 100% methanol for 30 min at RT and stained with crystal violet (1 g/100 mL) at RT for 15 min. Crystal violet was removed by aspiration, and the wells were washed gently with PBS until no more discoloration appeared. The stained cells were lysed in 200 µL 10% acetic acid for 10 min at RT, of which 150 µL was transferred to a 96‐well plate and the absorbance was measured at 595 nm (iMark Microplate Absorbance Reader, Bio‐Rad).

### 2.9 RNA interference

Transfection of *CD24* siRNA (Santa Cruz Biotechnology, Heidelberg, Germany) or scrambled RNA (‘AllStars Negative Control siRNA’, Qiagen, Hilden, Germany) has been performed as described previously [33]. Briefly, 1.5 × 10^5^ cells were transfected with FuGene HD (Promega, Mannheim, Germany) (5 µL FuGene HD: 1 µg siRNA). Formation of siRNA‐FuGene HD complexes was performed at RT for 15 min. Complexes were added slowly to the cells in standard culture medium without antibiotics. 24 h after transfection, cell culture medium was replaced with fresh standard medium with antibiotics.

### 2.10 DNA, RNA, and protein extraction

DNA was isolated by phenol/chloroform/isoamyl alcohol as published previously [34]. For RNA extraction, cells were harvested and RNA was isolated using the RNeasy Mini Kit (Qiagen) according to the manufacturer’s protocol. Concentrations and quality of isolate DNA/RNA were measured/checked by NanoDrop measurement (ratios 260/280 nm, 260/230 nm). Proteins were isolated by RIPA buffer as published [27]. Protein concentration was assessed by the ‘BCA Protein Assay Reagent Kit’ (Thermo Fisher Scientific).

### 2.11 DNA methylation analysis by sodium‐bisulfite sequencing

Analyses of DNA methylation patterns by sodium bisulfite sequencing or methylation‐specific PCR were performed as described previously [34]. 500 ng of genomic DNA was used for sodium bisulfite conversion (‘EZ DNA Methylation‐Gold’ Kit; Zymo Research, Freiburg, Germany). See Table S2 for oligonucleotides used for PCR (Table S2).

### 2.12 Sanger sequencing

For Sanger sequencing, PCR‐amplified DNA was cloned into the pCR2.1 vector using the ‘TA cloning kit’ (Thermo Fisher Scientific) according to the manual (ratio insert DNA to vector, 3:1). TOP10 *E. coli* bacteria were transformed according to the ‘TA cloning kit’ manual. Plasmid DNA was isolated from bacteria mini‐preps following an alkaline lysis protocol. An EcoR1‐HF restriction enzyme (New England Biolabs, Frankfurt a. M., Deutschland) double digest of 1 ug plasmid DNA for 30 min at 37 °C, followed by agarose gel electrophoresis, confirmed insertion of the PCR product into the plasmid. Plasmids were Sanger‐sequenced using M13 primers at Eurofins (Ebersberg, Germany).

### 2.13 Infinium MethylationEPIC BeadChip: sample preparation

Genomic DNA used for the Infinium MethylationEPIC BeadChip array (850 k) was isolated and purified by two rounds of PCI precipitation including a RNaseA treatment. DNA concentrations were measured by the ‘Qubit 4 Fluorometer’ (Thermo Fisher Scientific). The Infinium MethylationEPIC array BeadChip (Illumina, San Diego, CA, USA) was carried out by the Epigenomic Services from Diagenode (Liege, Belgium, Cat. nr. G02090000) following an established in‐house analysis pipeline. For each sample, 500 ng of DNA was used for sodium bisulfite conversion using the EZ‐96 DNA Methylation Kit (Zymo Research). Bisulfite conversion was controlled by qPCR. One assay targeting a methylated region of *DNAJC15* and two assays targeting the *GNAS* locus (one assay for the unmethylated allele and one assay for the methylated allele) were used for quality control. Deaminated DNA derived from blood was amplified in parallel and served as positive control. A sample passed the quality control when the received ct‐value either for the two *GNAS* loci or for the *DNAJC15* locus reaches the threshold not later than five cycles compared with the positive control.

### 2.14 Infinium MethylationEPIC BeadChip: array performance and data analysis

Data and bioinformatic analyses were carried out using ‘genomestudio’ software (version 2011.1; methylation module v1.9) (Illumina), and a detailed report on quality controls and internal control measurements provided by Diagenode is available upon request. Beta‐values were transformed into *M*‐values according to Du *et al*. [35]. Data analyses were performed as described previously [36, 37, 38]. Raw and processed DNA methylation data have been uploaded to ‘Gene Expression Omnibus’ (GEO) (GSE176450). A two‐group comparison was performed using the ‘qlucore omics explorer’ software version 3.7 by means of a *t*‐test to analyze for significance.

### 2.15 DNA dot blots

DNA dot blots for comparison of DNA methylation levels were performed as described and repeated twice [39]. Briefly, for each dot, 5 µL of a 1 : 2 dilution series of DNA (ranging from 500 to 62.5 ng diluted in H_2_O) was spotted onto a positively charged nylon membrane (Carl Roth GmbH + Co KG, Karlsruhe, Germany) and air‐dried for 15 min. Afterward, the membrane was UV‐cross‐linked (20 s, 1200 mJ·cm^−2^) and blocked in 5% milk powder in PBST for 1 h at RT. The membrane was incubated with 5mC antibody overnight at 4 °C and secondary antibody for 2 h at RT. Methylene blue‐stained membranes (0.04% methylene blue in 5 m sodium acetate) served as loading control. Signals were detected using the ChemiDoc Imaging Systems, and dot intensity was quantified using the ‘Image Lab’ software (both from Bio‐Rad). See Table S3 for antibody details.

### 2.16 CRISPR/Cas9‐mediated genome editing

Genome editing via CRISPR/Cas has been performed as published previously [24]. A PCR strategy was used to confirm successful CRISPR/Cas9 reaction; a primer pair was designed to amplify a DNA fragment of 160 base pairs (bp), which is case of successful deletion of parts of exons 1 and 2 (PCR product 1). A second primer pair was designed to amplify a ‘wild‐type’ sequence to screen for homo‐ or heterozygous *CD24* deficiency (PCR product 2, 234 bp). See Table S2 for guide RNA sequences.

### 2.17 Chromatin immunoprecipitation followed by quantitative PCR (ChIP‐qPCR)

ChIP‐qPCR was performed as published previously [40]. Briefly, 1 × 10^7^ cells were fixed for 30 min at RT using Diagenode ‘Crosslink Gold’ Kit (Diagenode). Cells were fixed 10 min in 1% formaldehyde (AppliChem, Darmstadt, Germany) and processed according to the ‘SimpleChIP Plus Enzymatic Chromatin IP’ Kit (Cell Signaling Technology, Leiden, The Netherlands). ChIP was carried out using 200 µg chromatin lysate and 5 µg antibody. 2% input (= 2 µg chromatin) and a ChIP using an IgG antibody served as controls. For ChIP‐qPCR, 10 µL of IP samples was amplified using the ‘Genome Plex Single Cell Whole Genome Amplification Kit’ (Sigma‐Aldrich) and subjected to qPCR at 1 : 40 dilution. Experiments were performed in triplicates. See Table S2 for used oligonucleotides and Table S3 for utilized antibodies.

### 2.18 Quantitative RT‐PCR

A total amount of 1 µg of RNA was *in vitro*‐transcribed, and qRT‐PCR was performed as described previously [27]. Gene expression was determined on the 384‐well C1000 cycler (Bio‐Rad). Each measurement was performed in triplicates. Utilized oligonucleotides are given in Table S2. *GAPDH* and *ACTB* were used as housekeeping genes and for data normalization.

### 2.19 RNA sequencing

RNA samples used for transcriptome analyses were quantified (Qubit RNA HS Assay, Thermo Fisher Scientific), and quality was measured by capillary electrophoresis using the Fragment Analyzer and the ‘Total RNA Standard Sensitivity Assay’ (Agilent Technologies Inc., Santa Clara, CA, USA). The library preparation was performed according to the manufacturer’s protocol (‘VAHTS Stranded mRNA‐Seq Library Prep Kit’). 300 ng total RNA was used for mRNA capturing, fragmentation, synthesis of cDNA, adapter ligation, and library amplification. Bead purified libraries were normalized and finally sequenced on the HiSeq 3000/4000 System (Illumina, Inc.) with a read setup of 1 × 150 bp. The bcl2fastq tool was used to convert the bcl files to fastq files as well for adapter trimming and demultiplexing. Data analyses on fastq files were conducted with clc genomics workbench (version 12.0.2; Qiagen). The reads of all probes were adapter (Illumina TruSeq) and quality‐trimmed (using the default parameters: Bases below Q13 were trimmed from the end of the reads, ambiguous nucleotides max. 2). Mapping was done against the *Homo sapiens* (hg38) (May 25, 2017) genome sequence. Statistical differential expression tests were determined using the ‘Differential Expression in two groups’ tool (version 1.02) (Qiagen). The resulting *P*‐values were corrected for multiple testing by false discovery rate (FDR) and Bonferroni correction. A *P*‐value ≤ 0.05 was considered significant. RNA‐seq data are freely available via GEO (GSE168646).

### 2.20 Illumina HT‐12v4/Affymetrix expression microarrays

Re‐analyses of our previously published Illumina and Affymetrix expression arrays of GCT cell lines (TCam‐2, *n* = 5; 2102EP, *n* = 5; NCCIT, *n* = 4; JAR, *n* = 2; FS1, *n* = 4; and MPAF, *n* = 4), SOX2‐deficient TCam‐2 cells *in vivo* (*n* = 5), *in vivo*‐reprogrammed TCam‐2 (*n* = 8), and GCT tissues (GCNIS, *n* = 3; seminomas, *n* = 4; Ecs, *n* = 3; and normal testis tissue, *n* = 4) were performed in the context of this study [26, 36, 41, 42, 43, 44]. Data are available via GEO (GSE71239, GSE71269, GSE79065, and GSE60698). For Affymetrix and Illumina microarrays, expression intensities of < 10 and < 7 were considered as ‘not expressed’, respectively. Thresholds were set based on the expression intensity of *SOX2* and *SOX17* in seminoma (*SOX2*−, *SOX17*+) and EC (*SOX2*+, *SOX17*−) tissues or cell lines.

### 2.21 Western blot

20 µg of whole protein lysates isolated by RIPA buffer was used for western blotting as described previously [36]. Beta‐ACTIN or VINCULIN was used as housekeeper and for data normalization. Utilized antibodies are listed in Table S3.

### 2.22 Mass spectrometry: sample preparation

Proteins were extracted from frozen cell pellets (NCCIT‐*CD24* ^+/+^ *n* = 3; NCCIT‐Δ*CD24 n* = 5) as described elsewhere [45]. Briefly, cells were lysed and homogenized in urea buffer with a TissueLyser (Qiagen) and supernatants were collected after centrifugation for 15 min at 14 000 ***g*** and 4 °C. Protein concentration was determined by means of Pierce 660 nm Protein Assay (Thermo Fischer Scientific), and 10 µg protein per sample was loaded on a SDS/PAGE for in‐gel digestion. The isolated gel pieces were reduced (50 µL, 10 mm DTT), alkylated (50 µL, 50 mm iodoacetamide), and underwent afterward tryptic digestion (6 µL, 200 ng trypsin in 100 mm ammonium bicarbonate). The peptides were resolved in 0.1% trifluoroacetic acid and subjected to liquid chromatography.

### 2.23 Mass spectrometry: LC‐MS analysis

For the LC‐MS analysis, a Q Exactive Plus (Thermo Fisher Scientific) connected with an Ultimate 3000 Rapid Separation Liquid Chromatography System (Dionex/Thermo Fisher Scientific) equipped with an Acclaim PepMap 100 C18 column (75 µm inner diameter, 25 cm length, 2 mm particle size from Thermo Fisher Scientific) was applied. The length of the LC gradient was 120 min. The mass spectrometer was operating in positive mode and coupled with a nano‐electrospray ionization source. Capillary temperature was set to 250 °C and source voltage to 1.5 kV. In the Q Exactive Plus mass spectrometer for the survey scans, a mass range from 350 to 2000 *m*/*z* at a resolution of 140 000 was used. The automatic gain control was set to 3,000,000, and the maximum fill time was 80 ms. The ten most intensive peptide ions were isolated and fragmented by high‐energy collision dissociation.

### 2.24 Mass spectrometry: data analysis, protein identification, and quantification

Mass spectrometric data were processed using the maxquant software version 1.6.17.0 (Max Planck Institute for Biochemistry, Planegg, Germany) applying standard parameters for label‐free protein identification and quantification. Searches were carried out based on 74811 Homo sapiens protein entries downloaded from the UniProtKB on March 27, 2020, using tryptic cleavage specificity (behind K and R) and a maximum of two missed cleavage sites. Carbamidomethylation at cysteine residues was considered as fixed modification; methionine oxidation and N‐terminal acetylation were considered as variable modifications. After an initial search using a precursor mass tolerance of 20 ppm and recalibration, a second search was performed with 4.5 ppm precursor mass tolerance. Tolerances for fragment spectra were 20 ppm. A FDR of 1% was used for both peptide and protein identification.

The intensity and LFQ intensity values received as output of the maxquant software were normalized, respectively, toward zero median log_2_(fold change) in sample pairwise comparisons of the LFQ intensities over all identified proteins. The sample exhibiting the highest LFQ intensities, leading to positive log_2_(fold change) medians in pairwise comparisons to all other samples before normalization, was used as reference sample; the LFQ intensities of the other samples were scaled by the respective factors, that is, 2^(median log2(fold change))^.

The normalized data set was filtered by importing the protein list into the perseus software version 1.6.6.0 (Max Planck Institute for Biochemistry) and removing potential contaminants, decoy hits, and proteins that were identified ‘by site’. Additionally, a minimum of three valid values had to be present in at least one group. The ‘Significance Analysis of Microarrays’ (SAM) method was applied on intensity and LFQ intensity values, separately [46]. LFQ intensities were log_2_‐transformed to reach a normal distribution like data structure, and missing values were filled in with random values from downshifted normal distribution (0.3 s. d. width, 1.8 s. d. downshift). Utilizing Student’s *t*‐test‐based SAM analysis, with a constant S_0_ of 0.17 (intensity values) or 0.12 (LFQ intensity values) and a 0.2 FDR‐based cut‐off, protein groups showing a significantly higher (or lower) abundance in the NCCIT‐Δ*CD24* samples with respect to the parental controls were referred to as up(or down)regulated proteins. As this method considers both the differences between the mean values of log_2_ (LFQ) intensities and the standard deviation of repeated measurements, proteins were considered as up‐ or downregulated proteins on the basis of abundance differences between NCCIT‐Δ*CD24* and parental cells and low *P*‐values. The final list of up‐ or downregulated proteins was obtained by combining the lists of the two separate SAM analyses (based on intensities or LFQ intensities) and filtering for the proteins that had Student's *t*‐test *P*‐value < 0.05 in both SAM analyses with the same direction of abundance change (upregulated in both analyses or downregulated in both analyses). The mass spectrometry proteomics data have been deposited to the ‘ProteomeXchange Consortium’ via the ‘PRIDE’ partner repository with the data set identifier PXD025110 [47].

### 2.25 Immunofluorescent staining

For immunofluorescent staining (IF) of CD24, 3 × 10^4^ cells per well were seeded onto 96‐well plates and incubated for 72 h. Cells were fixed by 4% formaldehyde for 10 min. Subsequently, cells were permeabilized for 5 min and blocked for 1 h at RT with 0.1% Triton X‐100 in PBS and 1.5% BSA in PBS, respectively. CD24 antibody was incubated at a dilution of 1 : 50 in blocking buffer overnight at 4 °C. The secondary antibody was added for 1 h in the dark at RT, and cells were counterstained with 0.5 μg·mL^−1^ 4′,6‐diamidine‐2′‐phenylindole dihydrochloride in PBS (DAPI, Sigma‐Aldrich) for 3 min. Antibody details are depicted in Table S3.

### 2.26 Immunohistochemistry

As published previously, immunohistochemical stainings were performed on 4‐μm formalin‐fixed and paraffin‐embedded tissue sections [48]. Antigen retrieval as carried out at 98 °C in EDTA buffer (pH 9; 20 min). Afterward, primary antibodies were incubated for 30 min at RT before incubation with a HRP‐labeled secondary antibody at RT for 25 min. Visualization of target antigen was performed by using ‘DAB+ Chromogen’ System (Dako, Hamburg, Germany). The experiments were undertaken with the understanding and written consent of each subject. Utilized antibodies are listed in Table S3.

### 2.27 Flow cytometry

Number of CD24^+^ cells were determined using a ‘MACSQuant’ flow cytometer with the ‘Flowlogic’ software (all from Miltenyi Biotec, Bergisch Gladbach, Germany). Cell suspensions were incubated with CD24‐FITC antibody (1 : 100) diluted in 100 μL FACS buffer (phosphate‐buffered saline (PBS), pH 7.2, 0.5% bovine serum albumin (BSA), and 2 mm EDTA) for 30 min in the dark at 4 °C. Afterward, cells were washed once with FACS buffer before analysis of at least 5 × 10^4^ cells. Utilized antibodies are listed in Table S3. Cell cycle analysis and apoptosis assay using PI or PI/Annexin V staining, respectively, with subsequent flow cytometric analysis have been performed as described previously [49].

### 2.28 CD24 antibodies

CD24 is a small and highly post‐translationally modified membrane protein, making detection of CD24 by antibody challenging [50]. Thus, in this study, we utilized three different antibodies (SWA11, SN3, and REA832) to detect CD24 on protein level by various methods, guaranteeing reliable results that back up each other [7, 50, 51].

### 2.29 Xenografting of TCam‐2 and 2102EP cells

Xenotransplantation of GCT cell lines has been performed as published previously [52]. Briefly, 1 × 10^7^ cells were injected in 500 µL Matrigel into CD1 nude mice using a G27 syringe. Animal experiments were performed under license of the ‘Landesamt für Natur und Umwelt‐Nordrhein‐Westfalen’ (LANUV‐NRW; AZ‐84‐02.04.2013‐A430).

### 2.30 Online analysis tools and software

Publicly available ‘The Cancer Genome Atlas’ (TCGA) data sets were analyzed using the ‘cBioPortal for Cancer Genomics’, the ‘UCSC Xena browser’, and ‘PINA’ [53, 54, 55, 56, 57]. The STRING algorithm was used to predict protein–protein interaction by confidence [58]. The DAVID algorithm has been used to predict molecular functions of deregulated genes/proteins found in RNA‐seq or mass spectrometry analyses based on ‘Gene Ontology’ (GO), ‘Kyoto Encyclopedia of Genes and Genomes’ (KEGG), UniProt, and INTEPRO [59]. ‘MethPrimer 2’ was used to design ‘primers’ for sodium bisulfite sequencing analyses and to screen for CpG dinucleotide density across the *CD24* gene. ‘Phyton’ was used to generate volcano and violin plots from DNA methylation, RNA‐seq, and MS data [60, 61]. ‘ClustVis’ was used to perform principal component analysis (PCA) of RNA‐seq data and the ‘Qlucore Omics Explorer’ to generate 3D‐PCA from DNA methylation data [62].

### 2.31 Statistical analyses

Differences between groups were analyzed using two‐tailed Student’s t‐test after confirming equality of two variances according to the F‐test. Statistically significant differences are highlighted by asterisk (*P < 0.05, *P < 0.05, or ***P < 0.005). Non‐significant differences are labeled by ‘n. s.’. Error bars are indicated by means of standard deviations (SDs).

## 3 Results

In this study, we characterized the expression of the signal transducer *CD24* in GCTs and further deciphered its molecular and (epi)genetic features.

### 3.1 Expression characteristics of *CD24*/CD24 in germ cell tumors

The gene coding for *CD24* is located on chromosome 6q21 and encodes for eight transcript variants (Fig. S1A). Non‐transcribed pseudogenes have been designated on chromosomes 1, 15, 20, and Y. The isoform *ENST00000606017.1*, which lacks exons 1, 3, and 4, is predominately found in GCTs, while the isoforms *ENST00000619133*.*4 (lacking parts of exon 1, and exons 2 and 4) and ENST00000619869.1* (lacking parts of exons 1 and 5, and exons 2, 3, and 4) can be found very rarely (Fig. S1A). In other CD24^+^ cancer entities (origin: bladder, prostate, kidney, skin, lung, liver, brain, and ovary), we found a similar expression profile with predominant expression of *ENST00000606017.1* and very weak expression of *ENST00000619133.4* (Fig. S1A).

**Fig. 1:**
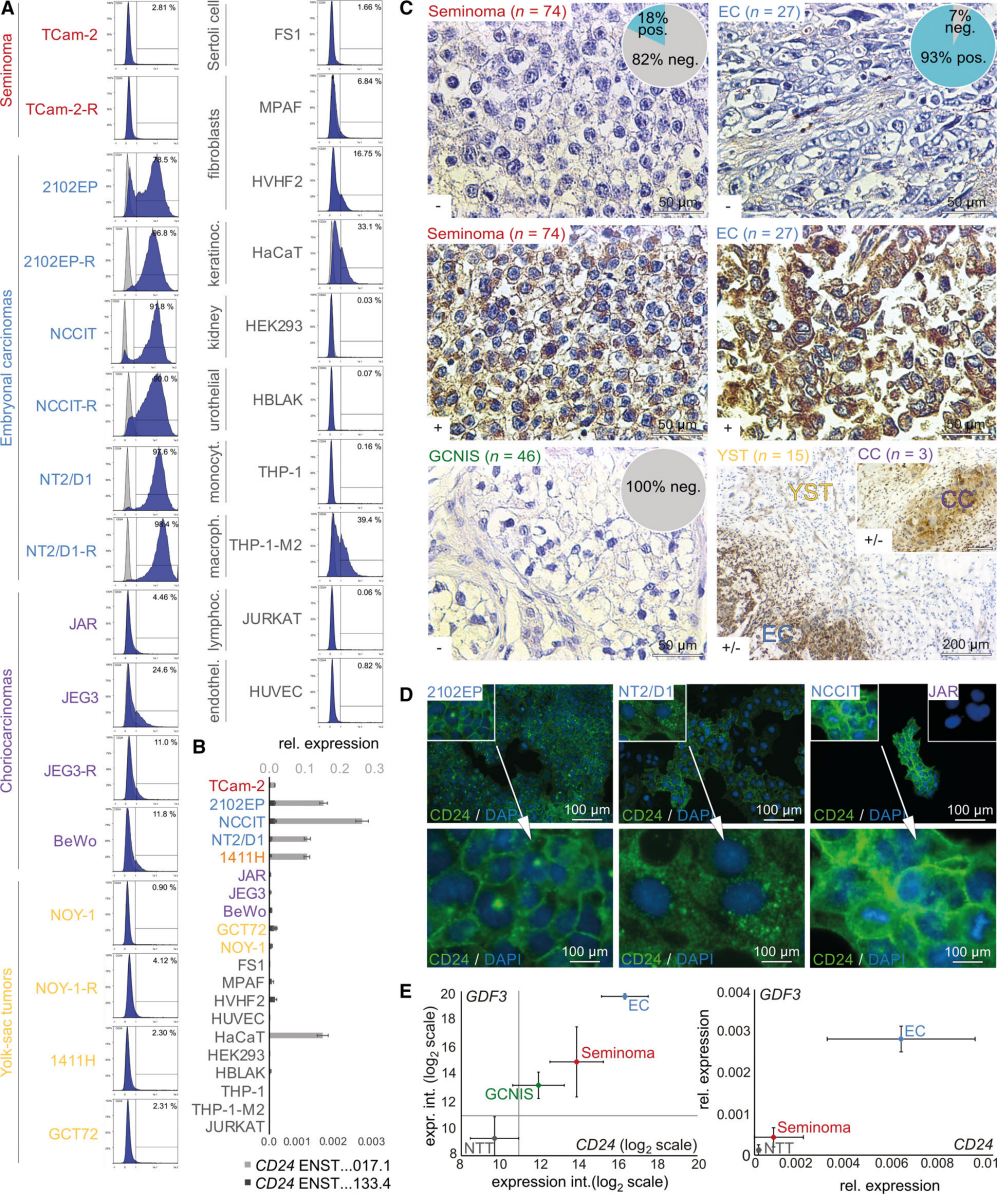
(A, B) Flow cytometry‐ and qRT‐PCR‐based analysis of CD24 protein (REA832 antibody) (A) and *CD24* mRNA (B) levels in indicated GCT cell lines and normal control cells (*n* = 3). As controls (gray), cells without addition of antibodies were measured. Error bars are indicated by means of standard deviations (SD). (C) Immunohistochemical staining of CD24 (SWA11 antibody) in GCT tissues. Scale bar = 50 µm; exception lower right corner incl. inlay = 200 µm. (D) Immunofluorescent staining of CD24 (SN3 antibody) in EC cell lines 2102EP, NCCIT, and NT2/D1. As a negative control, JAR cells were included. DAPI was used to stain nuclear DNA. Scale bar = 100 µm. (E) Expression microarray data (left) and qRT‐PCR analysis (right) of *CD24* and *GDF3* (as EC marker gene) in GCT tissues (GCNIS, seminoma, EC) and normal testis tissue as control. Error bars are indicated by means of standard deviations (SD).

We compared expression of *CD24* is a broad range of human malignancies by screening the ‘The Cancer Genome Atlas’ (TCGA) database. *CD24* is expressed in the majority of analyzed cancers (Fig. S1B). Based on CD24 expression status, GCTs appeared to be dichotomized into *CD24*‐high (RSEM > log_2_10) and *CD24*‐low (RSEM < log_2_10) tissues, arguing for a GCT subtype‐specific expression (Fig. 1B). Thus, we analyzed CD24 protein levels by flow cytometry in GCT cell lines including cisplatin‐resistant subclones (‐R) and normal healthy control cells (Fig. 1A). Specifically, in EC(‐R) cell lines, we found > 90% positivity for CD24. In the group of control cells, only keratinocytes, M2 macrophages (both < 40%), and fibroblasts (< 20%) showed CD24 positivity, but to level much weaker than in EC cells (Fig. 1A). We confirmed the flow cytometric data by analyzing *CD24* expression on mRNA level by qRT‐PCR (Fig. 1B). Again, we found high expression of the *CD24* isoform *ENST00000606017.1* in EC cells (2102EP, NCCIT, and NT2/D1). Although *CD24* is detected on mRNA level in TCam‐2, GCT72, and 1411H, only < 2.8% of the cells were positive for CD24 on protein level, suggesting for another layer of regulation of *CD24* mRNA processing (Fig. 1A,B). Expression intensity of the isoform *ENST00000619133.4* was negligibly low in all GCT cell lines and control cells (Fig. 1B, Fig. S1A). Additionally, in EC cells, the CD24 band patterning suggested different levels of post‐translational modifications, that is, glycosylation (Fig. S1C). Next, we stained GCNIS (*n* = 46), seminoma (*n* = 74), EC (*n* = 27), YST (*n* = 15), and CC (*n* = 3) tissues for CD24 by IHC (Fig. 1C; Fig. S1D). GCNIS was completely CD24^−^ (Fig. 1C; Fig. S1D). 93% of ECs were CD24^+^, while only 15% of seminoma cell components in mixed GCT setting and 18% of pure seminomas were CD24^+^ (Fig. 1C; Fig. S1D). YST and CC tissues presented as predominantly CD24^+^, with focal CD24^‐^ areas. Nevertheless, CD24 stained considerably weaker in YST (FOXA2^+^ [38]) and CC tissues compared with EC cells (OCT3/4^+^) (Fig. 1C; Fig. S1E). Of note, germ cells of different developmental stages (spermatogonia to sperm) were negative for CD24, which is in contrast to earlier findings in mice, which found a CD24^+^ spermatogonial population (Fig. S1F) [63]. CD24 stained strongly at the membrane and more weakly in the cytoplasm (Fig. 1C). This finding was confirmed by fluorescent staining of CD24 in EC cell lines 2102EP, NCCIT, and NT2/D1, which all showed CD24 positivity at the cell membrane and the cytoplasm (Fig. 1D). Additionally, we re‐analyzed expression microarray data and performed qRT‐PCR analyses of GCT tissues (GCNIS, seminomas, ECs) (Fig. 1E) [42]. As controls, normal testis tissues (NTT) were included. We correlated *CD24* expression to EC‐specific *GDF3* expression and demonstrated again that EC tissues show very high levels of *CD24* (and *GDF3*) expression (Fig. 1E).

### 3.2 Mutational burden of CD24 in germ cell tumors

Next, we screened the TCGA GCT cohort for mutations in the *CD24* gene locus (Fig. S1G). The vast majority of analyzed samples was extracted from testes, harbored the 12p gain, a high aneuploidy score, and was in the typical GCT age of 14–44 years at time point of diagnosis (Fig. S1G). No mutations of *CD24* were found in GCTs, suggesting that overexpression of *CD24* in ECs compared with the other GCT entities is not due to an amplification.

### 3.3 CD24 co‐detection with stem cell surface markers

**Fig. 2:**
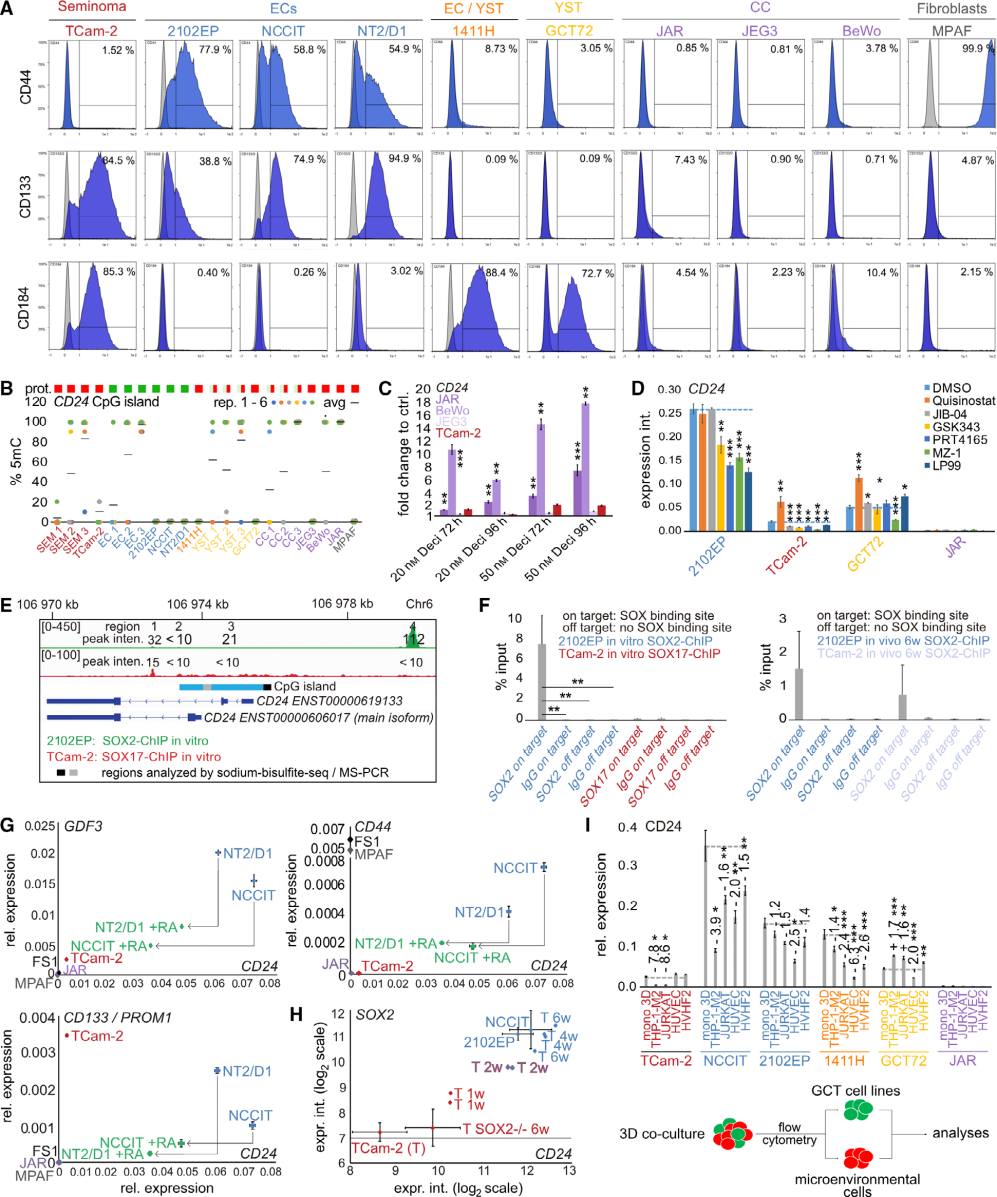
(A) Flow cytometric analysis of CD44, CD133, and CD184 (CXCR4) in GCT cell lines and MPAF fibroblasts. As controls (gray), cells without addition of antibodies were measured. (B) Sodium bisulfite sequencing analysis of the DNA methylation status of the *CD24* CpG island in GCT tissues and cell lines. Each sample has been analyzed in quintuplicates. (C) qRT‐PCR analysis of *CD24* expression in JAR, BeWo, JEG3, and TCam‐2 cells 72–96 h after 20 or 50 nm decitabine treatment (*n* = 3). Error bars are indicated by means of standard deviations (SD). (D) qRT‐PCR analysis of *CD24* expression in GCT and fibroblast cells treated for 16 h with related IC_50_ concentrations of the HDACi quisinostat, HDMi JIB‐04, HTMi GSK343, E3‐ULi PRT4165, and BRDi MZ‐1 and LP99. Error bars are indicated by means of standard deviations (SD). (E) SOX2‐ and SOX17‐ChIP‐seq in 2102EP (*n* = 3) and TCam‐2 (*n* = 3), respectively. Four putative SOX binding sites (region 1–4) were found within or in close proximity to the *CD24* coding sequence. The CpG island region analyzed by sodium bisulfite sequencing is labeled in light blue. (F) SOX2‐ and SOX17‐ChIP‐qPCR analysis in 2102EP or TCam‐2 cells, respectively. As control, an IgG antibody was used. For qPCR, two primers were used: (1) amplifying *CD24* region 4 (on target) and (2) amplifying a region with no SOX binding motif and not related to the *CD24* coding region (off target). Error bars are indicated by means of standard deviations (SD). (G) qRT‐PCR analysis of *CD24*, *GDF3, CD44,* and *CD133* expression in EC cell lines (NCCIT and NT2/D1) treated with 20 µm retinoic acid for 8 days. As controls and for comparison, other GCT and control cells were included (*n* = 3). Error bars are indicated by means of standard deviations (SD). (H) Expression microarray data of *CD24* and *SOX2* expression in GCT cell lines, SOX2‐deficient TCam‐2 grown *in vivo* for 6 weeks (T SOX2^‐/‐^ 6w) and during *in vivo* reprogramming of TCam‐2 into an EC‐like cell fate over 6 weeks (T 1w, T 2w, T 4w, and T 6w). (I) qRT‐PCR analyses of *CD24* expression in the GCT cell populations after co‐cultivation of GCT cell lines with immune cells (THP‐1‐M2 (M2 macrophages), JURKAT (T lymphocytes), endothelial cells (HUVEC), or fibroblasts (HVHF2)) for 72 h and flow cytometry‐based cell sorting (*n* = 3). Error bars are indicated by means of standard deviations (SD). Two‐tailed t‐tests were performed to test for significance; *P‐value < 0.05, **P‐value < 0.005, and ***P‐value < 0.0005.

CD24 in combination with CD44 and/or CD133 (PROM1) has been highlighted as cancer stem cell markers (in combination with *NANOG*, *OCT3/4*, and *SOX2)*, and a cell‐type‐dependent correlation of CD24 to the chemokine receptor CD184 (CXCR4) has been shown [64, 65, 66, 67, 68, 69, 70, 71, 72, 73, 74, 75]. As demonstrated by flow cytometric analysis, EC cell lines 2102EP, NCCIT, and NT2/D1 (NANOG^+^/OCT3/4^+^/SOX2^+^) presented as CD24^+^/CD44^+^/CD133^+^/CXCR4^‐^, seminoma‐like TCam‐2 (NANOG^+^/OCT3/4^+^/SOX17^+^) as CD24^‐^/CD44^‐^/CD133^+^/CXCR4^+^, YST cell lines 1411H and GCT72 (NANOG^‐^/OCT3/4^‐^/SOX2^‐^) as CD24^‐^/CD44^‐^/CD133^‐^/CXCR4^+^, and the CC cell lines JAR, JEG3, and BeWo (NANOG^‐^/OCT3/4^‐^/SOX2^‐^) as CD24^‐^/CD44^‐^/CD133^‐^/CXCR4^‐^ (Figs 1A and 2A). As a positive control, we included MPAF fibroblasts, which were highly positive for CD44 (99.9%).

### 3.4 Involvement of DNA methylation in regulation of *CD24* expression

We asked whether CD24 expression might be regulated by DNA methylation in GCTs. Thus, we performed sodium bisulfite sequencing of a region upstream the *CD24* transcription start site containing ten CpG dinucleotides and classified as a CpG island in GCT cell lines and tissues (Fig. S2A). In *CD24* ^‐^ CC cell lines, *CD24* methylation varied between 1.7% (JEG3), 50% (BeWo), and 100% (JAR), while all other cell lines analyzed harbored a strongly hypomethylated *CD24* CpG island (TCam‐2: 10%; 2102EP: 1.7%; NCCIT: 0%; NT2/D1: 0%; 1411H: 0%; GCT72: 0%; and MPAF fibroblasts: 0%) (Fig. 2B; Fig. S2B). Microdissected cells from seminoma, EC, YST, and CC tissues (*n* = 3) showed a sample‐dependent hypo‐ or hypermethylation (Fig. 2B, Fig. S2B). We validated these data in JAR, JEG3, and BeWo by performing PCR using a methylation‐specific (MS) primer and found a similar DNA methylation profile (Fig. S2C). Additionally, we analyzed CpG methylation around the alternative promoter region of the *CD24* main isoform (Fig. 2E, gray box) by MS‐PCR (Fig. S2B). Here, we also found the same DNA methylation profile as before, suggesting that the DNA methylation profile is comparable across the analyzed CpG island (Fig. S2B).

To correlate sample‐specific *CD24* DNA methylation to CD24 protein levels, we performed IHC of related GCT samples and found that DNA methylation levels did not correlate to CD24 protein levels (Fig. 2B, Fig. S2D). Within each sample, we found the *CD24* CpG island either fully methylated or completely demethylated, mimicking an imprinting‐like DNA methylation pattern (Fig. S2B).

To test whether demethylation of the *CD24* CpG island derepresses *CD24* gene expression, we treated CD24^‐^ and *CD24* CpG island hypermethylated JAR, hemimethylated BeWo, and unmethylated JEG3 (and TCam‐2) cells over 96 h with 20 or 50 nm of the DNA‐demethylating agent 5‐aza‐2’‐deoxycytidine (decitabine) and demonstrated a dose‐dependent induction of *CD24* expression only in cells harboring *CD24* hemi‐ or hypermethylation (JAR, BeWo) (Fig. 2C). By sodium bisulfite sequencing, we demonstrated that upregulation of *CD24* expression coincided with demethylation of the *CD24* CpG island in JAR cells 96 h after 20 (to 33%) and 50 nm (to 10%) decitabine treatment (Fig. S2E). These data suggest that at least in *CD24* hemi‐ or hypermethylated CC‐like cells, *CD24* expression could be influenced by demethylating the corresponding CpG island.

### 3.5 Effect of interfering with the epigenetic landscape on *CD24* expression

It has been shown that *CD24* expression can also be regulated on chromatin level, that is, by histone acetylation [76, 77]. Thus, we treated TCam‐2, 2102EP, GCT72, and JAR for 16 h with individual IC_50_ concentrations (Fig. S3A) of the histone deacetylase inhibitor (HDACi) quisinostat, resulting in histone H3 hyperacetylation (Fig. S3B) and upregulation of *CD24* in all cell lines showing negligible *CD24* mRNA/CD24 protein levels (TCam‐2, GCT27, and JAR) (Fig. 2D). In CD24^+^ 2102EP cells, HDAC inhibition had no effect on *CD24* expression (Fig. 2D), which we confirmed on protein level by western blotting (Fig. S3C). Additionally, we confirmed upregulation of CD24 upon quisinostat treatment (16 h, 5 nm) in JAR cells by western blotting (Fig. S3C). We extended and confirmed this finding by treating CD24^‐^ JEG3 and BeWo cells with quisinostat and using another HDACi (entinostat). Again, we found upregulation of *CD24* in JEG3, BeWo, TCam‐2, GCT72, and JAR, but not in 2102EP, after 16 h of treatment (Fig. S2D). How do other epidrugs, such as inhibitors against histone demethylases (HDMi; JIB‐04), histone methyltransferases (HMTi; GSK343), E3 ubiquitin ligases (E3‐ULi; PRT4165), and bromodomain‐containing proteins (BRDi, MZ‐1, and LP99), affect *CD24* expression in GCT cell lines (for IC_50_ concentrations, see Fig. S2E)? In 2102EP cells, a downregulation of *CD24* mRNA was detected after application of GSK323, PRT4165, MZ‐1, and LP99 (but not JIB‐04) (Fig. 2D). In TCam‐2 and GCT72, a considerable reduction in *CD24* expression was detected only after MZ‐1 application, while expression intensities in JAR cells were too low to be considered biologically relevant (Fig. 2D). Taken together, HDACi is able to induce *CD24* expression in CD24^‐^ GCT cells, while application of a HMTi, E3‐ULi, or BRDi strongly reduced *CD24* expression in CD24^+^ EC cells.

### 3.6 The role of SOX2 in regulating *CD24* expression

Next, we aimed at deciphering the transcriptional regulation of CD24 in GCTs. Since *CD24* expression can be induced by SOX2 in melanoma cells, we asked whether *CD24* might be regulated by SOX2 in ECs as well [78]. Thus, we first analyzed chromatin immunoprecipitation followed by sequencing (ChIP‐seq) data of SOX2 in the EC cell line 2102EP (SOX2^+^/CD24^+^) and found four SOX binding sites within or in close proximity to the genomic region coding for *CD24* (Fig. 2E, regions 1–4) [40]. Only one SOX binding site (region 4, peak intensity > 100) upstream of the *CD24* transcription start site (TSS) was bound by SOX2, suggesting that SOX2 transactivates *CD24* expression via binding to this region (canonical SOX2 motif: *TTTTCAGATGCAAAT*) (Fig. 2E). We also checked whether SOX17, which in part shows redundancy to SOX2 in regulating pluripotency in seminomas, binds to *CD24* as well [40]. In the SOX17^+^/SOX2^‐^/CD24^‐^ seminoma cell line TCam‐2, only negligible binding intensities of SOX17 to *CD24* could be detected (peak intensities < 15) (Fig. 2E). We verified binding of SOX2 to region 4 of *CD24* by performing ChIP‐qPCR analysis in 2102EP *in vitro* and *in vivo* (xenografted into nude mice; six weeks of growth) (Fig. 2F). Again, SOX17 did not bind to *CD24* region 4 in TCam‐2 cells, but upon *in vivo* reprogramming of TCam‐2 into an EC‐like cell fate (xenografted into nude mice; 6 weeks of growth; accompanied by *SOX2* up‐ and *SOX17* downregulation [36]), binding of SOX2 to *CD24* could be detected (Fig. 2F). Taken together, in GCTs SOX2, but not SOX17, seems to be able to transactivate *CD24* expression by binding to a region in proximity to the TSS.

Besides SOX2, GDF3 has been shown to stimulate *CD24* expression in melanoma cells [79]. Therefore, we treated GDF3^‐^/CD24^‐^ TCam‐2 and JAR cells with recombinant GDF3 (50, 100 nm; 48–72 h) (Fig. S3E). In both cell lines, no significant induction of *CD24* expression could be measured by qRT‐PCR, suggesting that GDF3 is not able to transactivate *CD24* expression in GDF3^‐^/CD24^‐^ GCT cells.

### 3.7 *CD24* expression dynamics during differentiation of EC cells and reprogramming of TCam‐2 into an EC‐like cell fate

We asked how *CD24* expression is affected during differentiation of EC cells and during reprogramming of seminoma‐like TCam‐2 cells into an EC‐like cell fate. First, we differentiated between NT2/D1 and NCCIT cells by all‐trans retinoic acid (RA; 20 µm, 8 days [41]) and screened for expression of *CD24*, *GDF3* (as EC marker)*, CD44*, and *CD133* (as EC stemness marker, Fig. 2A) (Fig. 2G). *CD24* expression decreased considerably upon RA treatment and coincided with downregulation of *GDF3*, *CD44,* and *CD133* (Fig. 2G). We analyzed microarray expression data of TCam‐2 cells grown over six weeks (w) *in vivo* and reprogrammed into an EC‐like cell fate (T 1w‐6w, in duplicates) and *in vivo*‐grown *SOX2*‐deficient TCam‐2 cells (maintain a seminoma cell fate) [36, 41, 42, 44] (Fig. 2H). During *in vivo* reprogramming of TCam‐2 cells, *CD24* (and *SOX2*) became strongly upregulated over time to expression levels comparable to EC cell lines 2102EP and NCCIT (Fig. 2H). In contrast, *CD24* (and *SOX2*) expression remained low in *in vivo*‐grown SOX2‐deficient TCam‐2 cells (Fig. 2H). In summary, *CD24* expression can be associated with an undifferentiated EC cell fate and a naïve pluripotency status and correlates to *SOX2* expression.

### 3.8 The influence of the somatic microenvironment on *CD24* expression

CD24 is an important factor in the crosstalk of tumor cells with the somatic microenvironment [80, 81, 82, 83]. Specifically, a strong correlation between the phenotypic plasticity of a tumor, driven, for example, by epithelial‐to‐mesenchymal transition (EMT), and cues from the tumor microenvironment, eventually leading to cellular reprogramming, has already been postulated [84]. In hepatocellular and ovarian carcinoma, a link between EMT and high CD24 protein levels has been described [11, 85, 86]. Thus, we asked how *CD24* expression is affected in GCT cells after three‐dimensional co‐cultivation with M2 macrophages (differentiated THP‐1 cells; THP‐1‐M2 [31]), T lymphocyte‐like cells (JURKAT), endothelial cells (HUVEC), and fibroblasts (HVHF2). Due to the phenotypic plasticity, we expected that co‐culture with microenvironmental cells could result in changes of the pluripotency and/or differentiation status in GCT cells, eventually resulting in a reduction in *CD24* mRNA expression. Therefore, we co‐cultivated GCT cell lines showing *CD24^mRNA^ ^high^* (NCCIT, 2102EP), *CD24^mRNA^ ^low^* (TCam‐2, 1411H, GCT72), and *CD24^mRNA negative^* (JAR) cells with microenvironmental cells as hanging drops for 72 h. To be able to sort the different cell populations after co‐cultivation by flow cytometry, we generated GFP^+^ GCT and mCherry^+^/DeepRed^+^ microenvironmental cells (Fig. S3F). Purity of isolated cell populations was demonstrated by qRT‐PCR analysis of *DCN* (fibroblast marker), *CD36* (M2 macrophage marker), *CD6* (T‐lymphocyte marker), and *CD31*/*PECAM‐1* (endothelial cell marker) expression; *DCN*, *CD36, CD6,* and *CD31* expression was only detectable in HVHF2, THP‐1‐M2, JURKAT, or HUVEC cell populations, respectively (Fig. S3G). Upon co‐cultivation with THP‐1‐M2 or JURKAT immune cells, *CD24* expression was downregulated in TCam‐2, NCCIT, 2102EP, and 1411H, while *CD24* expression was slightly upregulated in GCT72 cells (Fig. 2I). Co‐cultivation with HUVEC endothelial cells reduced *CD24* expression specifically in non‐seminomatous cell lines NCCIT, 2102EP, 1411H, and GCT72, while co‐cultivation with HVHF2 fibroblasts reduced *CD24* expression in NCCIT, 2102EP, and 1411H cells (Fig. 2I). In JAR cells, *CD24* expression remained negligibly low under all conditions (Fig. 2I). Thus, upon crosstalk of GCT cells with the somatic microenvironment, especially immune cells (T cells, M2 macrophages), *CD24* expression might become downregulated.

### 3.9 Deciphering the molecular function of CD24 in EC cells

**Fig. 3:**
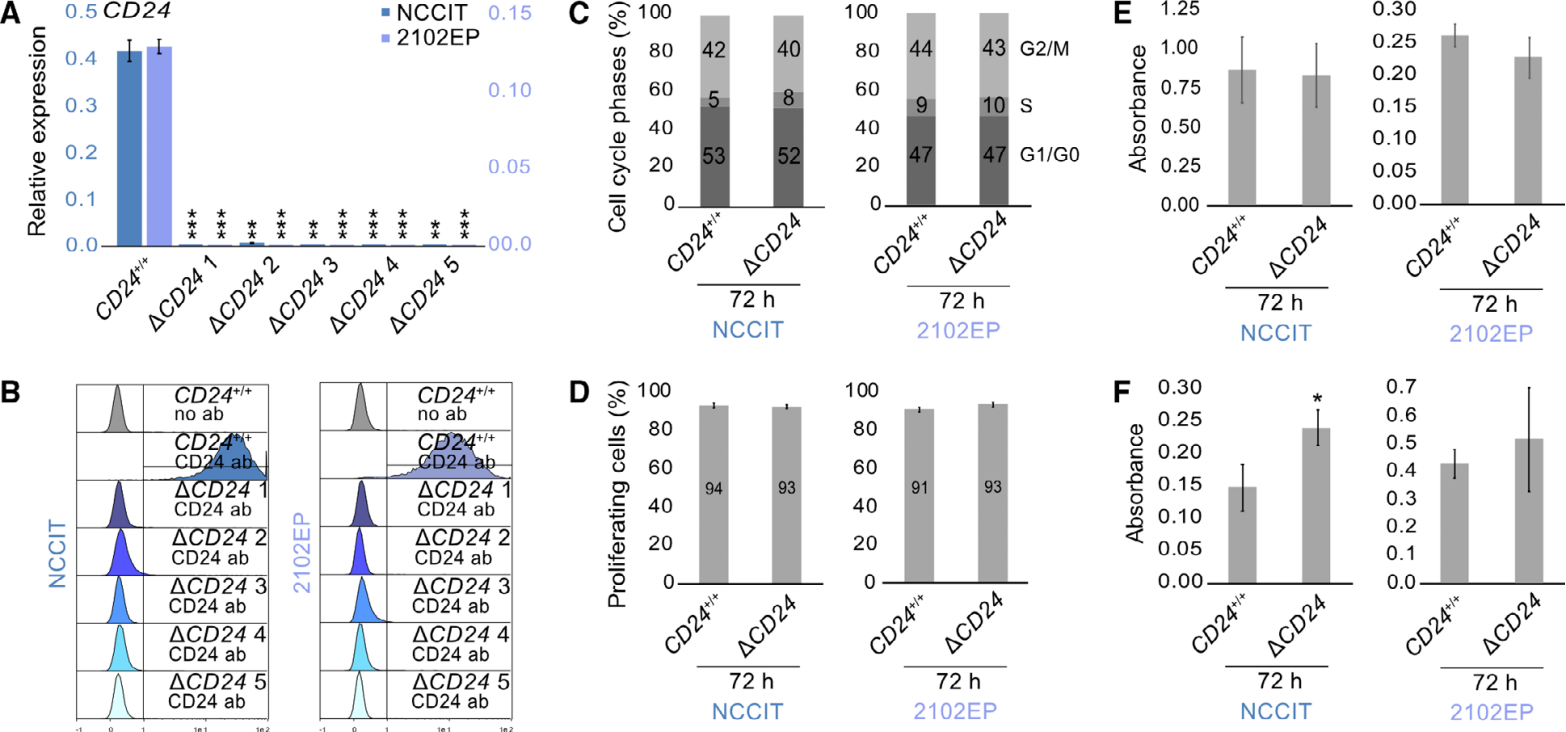
(A, B) qRT‐PCR (A) (*n* = 3) and flow cytometry (B) (NCCIT‐*CD24* ^+/+^ *n* = 1; NCCIT‐Δ*CD24 n* = 5) analysis of *CD24* expression and CD24 protein levels (REA832 antibody) in NCCIT‐ and 2102EP‐Δ*CD24* clones and parental cells. Ab = antibody. Error bars are indicated by means of standard deviations (SD). (C, D) Flow cytometry‐based measurement of cell cycle phase distribution (C) and proliferation rates (D) in NCCIT‐ and 2102EP‐Δ*CD24* clones and parental cells 72 h after plating (NCCIT‐*CD24* ^+/+^, *n* = 2; NCCIT‐Δ*CD24*, *n* = 5). Error bars are indicated by means of standard deviations (SD). (E, F) Measurement of adhesion ability (E) and migratory capacity by transwell assays (F) and in NCCIT‐ and 2102EP‐Δ*CD24* clones and parental cells 24 h after plating (NCCIT‐*CD24* ^+/+^, *n* = 3; NCCIT‐Δ*CD24*, *n* = 5). Error bars are indicated by means of standard deviations (SD). Two‐tailed t‐tests were performed to test for significance; *P‐value < 0.05, **P‐value < 0.005, and ***P‐value < 0.0005.

To decipher the molecular function of CD24 in more detail, we generated *CD24*‐deficient NCCIT and 2102EP EC cell lines (*n* = 5) by the CRISPR/Cas9 method (Fig. S4A,B). In *CD24*‐deficient clones (−Δ*CD24*), *CD24*/CD24 was strongly reduced compared with parental cells (−*CD24* ^+/+^) as demonstrated by qRT‐PCR, flow cytometric, and western blot analyses (Fig. 3A,B; Fig. S4C).

In contrast to data gathered in breast cancer cells, the cell cycle profiles, proliferation rates, and the adhesion abilities were not different between *CD24*‐deficient and parental cells, but the migratory capacity trended to be increased (Fig. 3C–F) [87, 88].

**Fig. 4:**
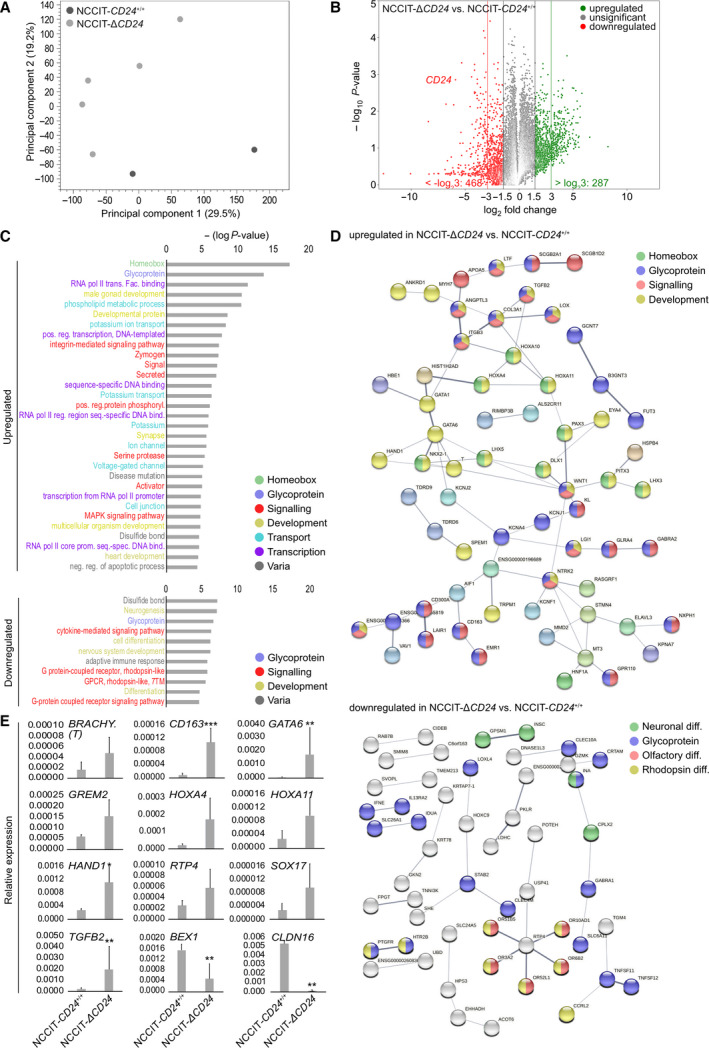
(A) PCA of RNA‐seq data of NCCIT‐Δ*CD24* clones and parental cells (NCCIT‐*CD24* ^+/+^, *n* = 2; NCCIT‐Δ*CD24*, *n* = 5). (B) Illustration of differentially expressed genes in NCCIT‐Δ*CD24* cells compared with the parental cells. (C) DAVID‐based prediction of biological processes and molecular functions in which the genes deregulated in NCCIT‐Δ*CD24* cells compared with the parental cells are involved in. (D) STRING‐based protein interaction prediction of the molecules upregulated or downregulated in NCCIT‐Δ*CD24* cells compared with the parental cells. (E) qRT‐PCR validation (*n* = 3) of selected deregulations in gene expression found by the RNA‐seq analysis in NCCIT‐DCD24 and parental cells. Error bars are indicated by means of standard deviations (SD). Two‐tailed *t*‐tests were performed to test for significance; *P‐value < 0.05, **P‐value < 0.005, and ***P‐value < 0.0005.

We performed RNA‐seq of NCCIT‐Δ*CD24* (*n* = 5) and parental cells (*n* = 2) to screen for differences in the transcriptome caused by *CD24* deficiency (Table S4A). Only RNA samples with a RNA quality value of > 9 were used (Fig. S4D). A principal component analysis (PCA) demonstrated that NCCIT‐Δ*CD24* cells clearly clustered apart from parental cells (Fig. 4A). On mRNA level, we detected 287 genes upregulated (representing genes negatively regulated by CD24) and 465 genes downregulated (representing genes positively regulated by CD24) in NCCIT‐Δ*CD24* cells versus parental cells (fold change (FC) > log_2_3) (Fig. 4B; Table S4B). *CD24* was among the set of genes strongly downregulated in NCCIT‐Δ*CD24* cells (FC: ‐ log_2_5.3) (Fig. 4B). By the STRING and DAVID algorithm, we predicted interaction and biological processes/functions of deregulated genes, respectively (Fig. 4C,D; Table S4B). The genes upregulated in NCCIT‐Δ*CD24* cells are homeobox or glycoproteins involved in the regulation of transcription and protein processing, potassium transport, and signaling processes controlling development/differentiation (via MAPK pathway) (Fig. 4C,D). Regarding differentiation, in detail genes associated with germ cell/spermatogenesis (*BRDT*, *GATA1*/*6*, *FSHR*, *HOXA10*/*11*, *SPEM1*, *TDRD6*/*9*, *TGFB2*), limb (mesoderm; *HNF1A*, *HOXA10*/*11*, *TGFB2*), heart (mesoderm; *GATA1*, *COL3A1*, *HAND1*, *LOX*, *TGFB2*), uterus (mesoderm; *HOXA10*/*11*, *TGFB2*), and skeletal (mesoderm; *COL3A1*, *HOXA10*/*11*, *MYOD1*, *TGFB2*) development were upregulated (Table S4B). Genes/glycoproteins downregulated in NCCIT‐Δ*CD24* cells could be associated with neuronal (ectodermal; *EZF1*, *GPSM1*, *BEX1*, *CPLX2*, *INSC*, *INA*, *LINC01587*, *NEUROG3*, *NRSN1*) and olfactory/eye‐related (ectodermal; *HTR2B*, *CCRL2*, *MRGPRG*, *ACKR2*, *OR10AD1*, *OR3A2*, *OR51B5*, *OR52L1*, *OR6B2*, *PTGFR*, *RGR*) differentiation processes (via G‐coupled receptors) (Fig. 4B,D). We validated selected deregulations in gene expression by qRT‐PCR and confirmed upregulation of *BRACHYURY (T)*, *CD163*, *CLDN16*, *GATA6*, *GREM2*, *HOXA4/11*, *HAND1, RTP4, SOX17,* and *TGFB2,* as well as downregulation of *CLDN16* and *BEX1* (Fig. 4E). Additionally, we performed a short‐time siRNA‐mediated knockdown of *CD24* expression in NCCIT cells, resulting in a considerable decrease in the CD24 protein level 48 h after transfection (Fig. S4E). By qRT‐PCR analysis, we validated downregulation of *CGB3*, *CLDN16,* and LIN28, upregulation of *HAND1*, *HOXA11,* and *GATA6,* and unchanged expression of *NANOG* and *OCT3/4* as found by the RNA‐seq analysis (Fig. S4E; Table S4A). Furthermore, by using liquid chromatography paired with mass spectrometry (LC‐MS), we took a proteome snapshot of NCCIT‐Δ*CD24* (*n* = 5) and parental cells (*n* = 3) (Table S4C). A PCA demonstrated that NCCIT‐Δ*CD24* cells clearly clustered apart from parental cells (Fig. S5A). 163 proteins were significantly enriched in the proteome of NCCIT‐Δ*CD24* compared with NCCIT‐CD24^+/+^ cells, and 99 were depleted (> FC 2) (Fig. S5B; Table S4D). By utilizing the STRING and DAVID algorithm again, we demonstrated that these proteins are involved in similar processes as found when analyzing the RNA‐seq data (Fig. S5C,D; Table S4D). Additionally, in the sets of enriched and depleted proteins, we found proteins associated with chromatin and protein modifications, such as acetylation, methylation, ubiquitination, and citrullination (Fig. S5C,D, Table S4D).

### 3.10 Correlation of gene expression dynamics to alterations in DNA methylation

**Fig. 5:**
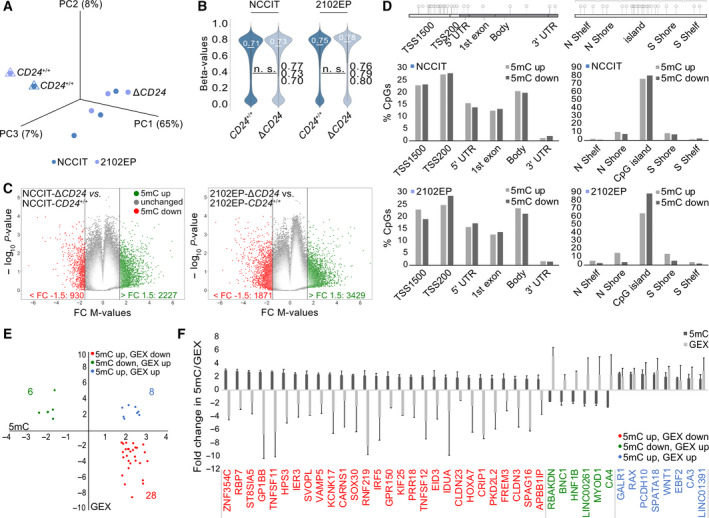
(A) PCA of 850 k DNA methylation array data in NCCIT‐ and 2102EP‐Δ*CD24* clones and parental cells (NCCIT‐*CD24* ^+/+^, *n* = 1; NCCIT‐Δ*CD24*, *n* = 3). (B) Violin plots illustrating global DNA methylation levels in NCCIT‐ and 2102EP‐Δ*CD24* clones and parental cells. Global DNA methylation levels of the individual *CD24*‐deficient clones are given on the right side of each bar (n. s. = not significant). (C) Illustration of differentially methylated genes in NCCIT‐/2102EP‐Δ*CD24* cells compared with the parental cells. A two‐group comparison (*t*‐test) was performed to sort for significance. (D) Affected by DNA methylation gene and CpG island regions in NCCIT‐/2102EP‐Δ*CD24* cells compared with the parental cells. Illustrations were taken from the ‘Illumina Infinium HumanMethylation450 BeadChip’ datasheet. (E, F) Volcano plot (E) and waterfall diagram (F) of commonly deregulated genes in NCCIT‐/2102EP‐Δ*CD24* cells showing inverse correlation between DNA methylation (5mC) and gene expression (GEX). Error bars are indicated by means of standard deviations (SDs).

Our previous data demonstrated a link between CD24 and the epigenetic landscape. Therefore, we analyzed changes in DNA methylation (5mC) in *CD24*‐deficient NCCIT and 2102EP cells compared with parental controls by Infinium MethylationEPIC bead arrays (Fig. 5, Table S4E). A PCA demonstrated that NCCIT‐/2102EP‐Δ*CD24* cells clearly clustered apart from parental cells (Fig. 5A). Global DNA methylation levels increased only slightly in NCCIT/2102EP‐Δ*CD24* cells compared with parental controls (NCCIT: + 2%; 2102EP: + 3%) (Fig. 5B). We confirmed this increase in 5mC levels by DNA dot blotting using a 5mC antibody (Fig. S4F). We identified 3157 differentially methylated CpG dinucleotides (2227 5mC up; 930 5mC down; ΔM‐value: 1.5) in NCCIT (Δ*CD24* vs. control) and 5300 in 2102EP (3429 5mC up; 1871 5mC down) (Fig. 5C; Table S4E). Changes in DNA methylation of these differentially methylated CpGs occurred mainly in CpG island context and around transcription start sites (TSS220, TSS1500) (Fig. 5D). 163 CpG dinucleotides were commonly altered in *CD24*‐deficient NCCIT and 2102EP cells (104 5mC up; 59 5mC down) (Table S4C). In NCCIT cells, we correlated changes in 5mC to changes in gene expression (GEX) found by the RNA‐seq analysis (Fig. 5E,F; Table S4F). For subsequent analysis, we only considered genes linked to a known ‘gene symbol’ and with significant alterations in DNA methylation (difference in *M*‐values > 1.5) in at least three CpG dinucleotides in all three *CD24*‐deficient samples and with a significant difference in gene expression of FC > log_2_1.5 (Table S4F). In the *CD24*‐deficient cells, we found 6 genes that were upregulated in expression and showed a decrease in 5mC (Group 1) and 28 genes that were downregulated in expression and showed an increase in 5mC (Group 2) (Fig. 5E,F). Additionally, we identified genes upregulated in expression and showing an increase in DNA methylation (Group 3), but found no gene showing downregulation in expression and a decrease in DNA methylation (Fig. 5F, Table S4F). Among Group 1 (5mC down, GEX up) were differentiation factors such as *MYOD1* and *HNF1A* and the zinc finger protein basonuclin‐1 coding gene *BCN1*, which is highly abundant in germ cells (Figs 5F and 4D). Thus, we postulate that expression of these genes might be influenced by DNA methylation. Among Group 2 (5mC up, GEX down) were previously identified STRING interactors such as HPS3, *IDUA*, *SVOPL,* and *TNFSF11/12*, as well as two claudins (*CLDN3/23*) (Figs 5F and 4D).

### 3.11 The effects of *CD24* deficiency on differentiation of EC cells

Analyses of the *CD24*‐deficient NCCIT cells pointed at an involvement of CD24 in poising the cells for ectodermal differentiation and blocking mesodermal differentiation.

**Fig. 6:**
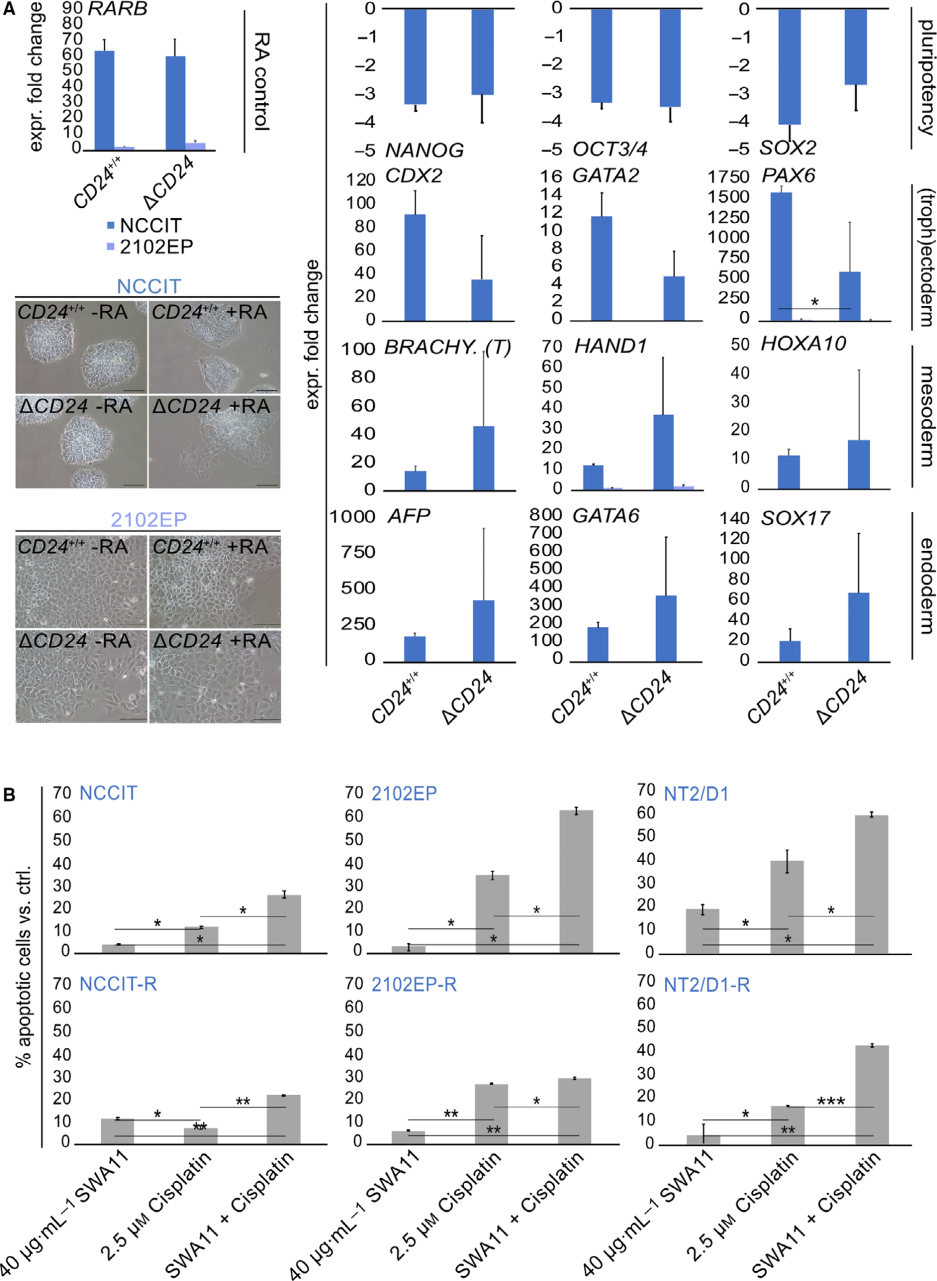
(A) qRT‐PCR analysis (*n* = 3) of indicated marker genes of all three germ layers ((extra‐embryonic (exe))‐endo‐, meso‐, ectoderm) after 10 days of RA‐mediated differentiation of NCCIT‐ and 2102EP‐Δ*CD24* and parental cells (NCCIT‐*CD24* ^+/+^, *n* = 1; NCCIT‐Δ*CD24*, *n* = 5). Error bars are indicated by means of standard deviations (SD). Additionally, exemplary pictures of cell morphologies +/− RA are given. (B) Flow cytometry‐based measurement (*n* = 3) of apoptosis rates after application of SWA11 and/or cisplatin in EC cell lines and corresponding cisplatin‐resistant subclones (‐R). Error bars are indicated by means of standard deviations (SD). Two‐tailed t‐tests were performed to test for significance; *P‐value < 0.05, **P‐value < 0.005, and ***P‐value < 0.0005.

Thus, we asked how RA‐mediated differentiation is altered in pluripotent and RA‐sensitive NCCIT‐Δ*CD24* cells compared with the parental cells. Therefore, cells were treated with RA (20 µm) for ten days and we analyzed changes in gene expression by qRT‐PCR afterward (Fig. 6A). Besides changes in the morphological appearance, a strong upregulation of *RARB* and downregulation of the pluripotency factors *NANOG*, *OCT3/4*, and *SOX2* in NCCIT‐Δ*CD24* and parental cells indicated a successful RA treatment (Fig. 6A) [41]. Mesodermal (*BRACHYURY (T)*, *EOMES*, *HAND1*) and endodermal (*AFP*, *GATA6*, *SOX17*) genes were more strongly upregulated, while ectodermal genes (*CDX2*, *GATA2, PAX6*) were more downregulated in NCCIT‐Δ*CD24* compared with the NCCIT‐*CD24* ^+/+^ cells (Fig. 6A). We also included nullipotent 2102EP cells, but as expected, RA application did not induce differentiation, indicated by an unchanged morphology and expression of genes indicative of a successful RA treatment, that is, *RARB*, *PAX6,* and *HAND1* (Fig. 6A) [41]. These findings strengthen our hypothesis that CD24 plays a role in cellular differentiation processes and is involved in blocking mesodermal and endodermal differentiation and poising pluripotent EC cells toward ectodermal differentiation (Fig. 6A).

### 3.12 Blocking CD24 as a new therapeutic option to render EC cells that are cisplatin‐sensitive

Finally, we analyzed whether blocking CD24 by the SWA11 antibody affects efficacy of cisplatin application in EC cell lines including cisplatin‐resistant subclones (‐R) [51]. By flow cytometry‐mediated measurement of apoptosis rates, we demonstrated that blocking CD24 considerably increased efficacy of cisplatin in all tested cell lines, while application of the SWA11 antibody alone had only minor effects (Fig. 6B). In conclusion, targeting CD24 in combination with cisplatin might be a reasonable concept to treat (cisplatin‐resistant) CD24^+^ GCT cells.

## 4 Discussion

In this study, we characterized the molecular and epigenetic mechanisms regulating the signal transducer and glycoprotein CD24 in GCTs.

**Fig. 7:**
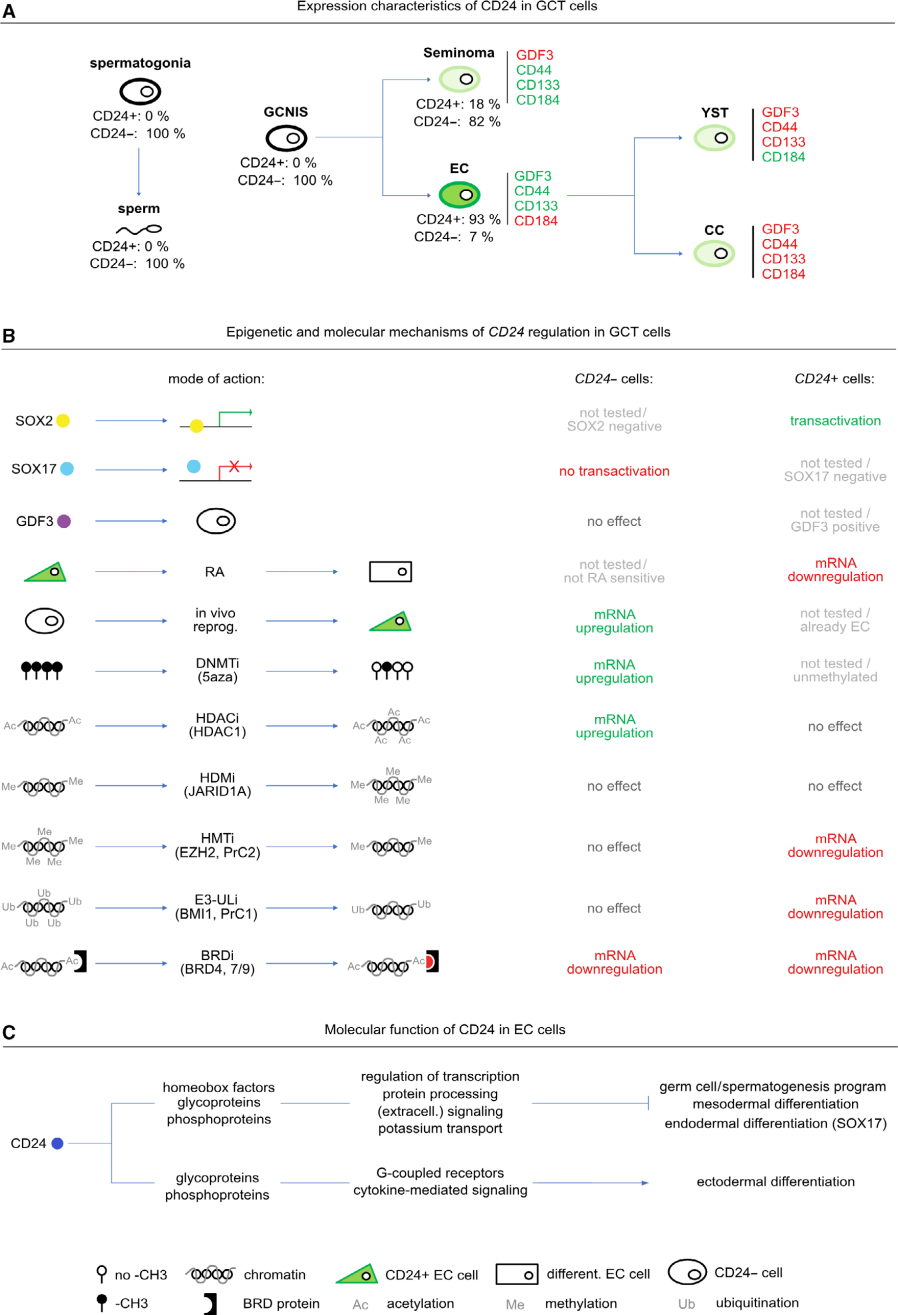
(A–C) Models summarizing key findings of this study. (A) *CD24* expression characteristics and dynamics in different GCT entities. (B) Molecular and (epi)genetic mechanisms regulating or influencing *CD24* expression. (C) Overview of the molecular function of *CD24* in ECs.

Overexpression of CD24 has been found in various cancers, such as lung, breast, and ovarian [9]. In GCTs, *CD24* (isoform *ENST00000606017.1)* is strongly expressed in ECs and absent to very weakly expressed in germ cells of different developmental stages, seminomas, YSTs, and CCs (Fig. 7A). GCNIS were completely CD24^‐^, suggesting that induction of *CD24* occurs mainly during reprogramming of GCNIS into an EC and is downregulated again upon differentiation into non‐seminomatous subentities, which is in line with strong downregulation of *CD24* in RA‐treated EC cells (Fig. 7B). Thus, in ECs, *CD24* expression can be associated with an undifferentiated and pluripotent cell fate.

A correlation of *CD24* to stemness markers *CD44*, *CD133* (*PROM1*), and *CD184* (*CXCR4*) has been demonstrated in various tumor types with breast cancer stem cells being CD24^‐^ and CD44^+^, while pancreatic, cholangiocarcinoma, colorectal, gastric, and multiple myeloma cancer stem cell‐like cells are CD24^+^/CD44^+^ [64, 65, 66, 67, 68, 69, 70, 71]. Hepatocellular and renal carcinomas are CD24^+^ and CD133^+^ [72, 73]. Additionally, a cell‐type‐dependent correlation of CD24 to the chemokine receptor CD184 (CXCR4) was found; for example, in pre‐B lymphocytes, CD24 negatively regulated *CD184*, while in cholangiocarcinomas, CD24 induced *CD184* expression [74, 75]. In GCTs, each analyzed entity displayed an individual marker expression profile, with ECs mainly presenting as CD24^strong^/CD44^+^/CD133^+^/CD184^‐^, seminomas as CD24^‐^/CD44^+^/CD133^+^/CD184^+^, YSTs as CD24^weak^/CD44^‐^/CD133^‐^/CD184^+^, and CCs as CD24^weak^/CD44^‐^/CD133^‐^/CD184^‐^ (Fig. 7A).

How is *CD24* regulated in GCTs? We demonstrated that SOX2 binds to a SOX motif in proximity to the *CD24* transcription start site and transactivates *CD24* in ECs (Fig. 7A). In line, Vencken *et al*. [89] demonstrated that a RNAi‐mediated knockdown of *SOX2* in 2102EP cells led to a 3.3‐fold downregulation of *CD24* expression. Additionally, SOX2 has already been shown to induce *CD24* expression in a melanoma cell or a murine model [78, 79, 90, 91].

So, SOX2 is a *bona fide* transactivator of *CD24* expression in ECs. In our RNA‐seq analysis and related qRT‐PCR validation, *SOX17* was upregulated 3.7‐fold in NCCIT‐Δ*CD24* cells compared with the parental clones (Table S4A; Fig. S4D). Additionally, SOX17 became strongly upregulated in RA‐treated NCCIT‐Δ*CD24* cells compared with RA‐treated parental cells (Fig. 6A). Thus, CD24 might be involved in suppressing *SOX17* expression in ECs.

Our results suggest an involvement of epigenetic mechanisms in the regulation of *CD24*. On the one hand, we found sample‐dependent and highly variable *CD24* DNA methylation patterns, which did not correlate to *CD24* mRNA or CD24 protein levels. On the other hand, *CD24* expression could be derepressed upon decitabine‐mediated DNA demethylation in CC cell lines, suggesting that at least in choriocarcinomas, DNA methylation might be involved in silencing *CD24*. Transcriptional regulation of *CD24* independent of the DNA methylation status has been described in breast cancers [77]. Thus, DNA methylation seems to play a minor role in regulation of *CD24* expression in GCTs. CD24 itself influences the DNA methylation landscape only slightly, but induces site‐specific changes in DNA methylation of factors deregulated in *CD24*‐deficient EC cells, such as demethylation of differentiation‐ and germ cell‐related factors such as *MYOD1* and *BNC1*.

Our data point at histone acetylation as an additional regulator of *CD24*, since application of HDACi led to histone hyperacetylation, resulting in *CD24* upregulation in CD24^‐^ GCT cell lines. In CD24^+^ EC cells, where *CD24* expression remained unaffected upon HDACi application, *CD24* expression was downregulated upon treatment with inhibitors of histone methyltransferases (HMTi), E3 ubiquitin ligases (E3‐Uli), or histone‐code readers (BRDi), indicating that histone methylation or the polycomb repressive complex may be required for proper *CD24* expression in ECs (Fig. 7B).

Some GCT cell lines (TCam‐2, 1411H, GCT72) show *CD24* expression on mRNA level, but do not translate CD24 protein. Here, we propose that *CD24* mRNA is bound by microRNAs (miR), thereby preventing initiation of translation. Regulation of *CD24* by *miR34a, miR‐1185‐1,* and *miR146a* has already been proposed in colon and breast cancer cells and oral squamous cell carcinomas [92, 93, 94].

We also found that three‐dimensional interaction of CD24^+^ EC cells with fibroblasts, and endothelial or immune cells is able to reduce *CD24* expression. Thus, interaction of EC cells with the somatic microenvironment, for example, during metastasis, might lead to *CD24* downregulation, putatively sensitizing the cells to differentiation. Our results indicate that mainly the interaction with immune cells (T cells, M2 macrophages) leads to *CD24* downregulation. Thus, triggering the immune system by immune‐modulating therapy might also lead to *CD24* downregulation, which in turn may render the tumor cells more sensitive toward cisplatin (Fig. 6B). In conclusion, combining immunotherapy with CD24 blocking agents could be a promising therapeutic approach to re‐sensitize chemotherapy‐resistant tumors toward cisplatin‐based therapy.

By establishing *CD24*‐deficient EC cell clones, we demonstrated that CD24 could be involved in blocking the germ cell/spermatogenesis program and mesodermal differentiation in ECs (Fig. 7C). Among the genes upregulated in NCCIT‐Δ*CD24* cells are many homeobox or glycoproteins involved in regulation of transcription and protein processing, suggesting that CD24 interacts with homeobox and glycoproteins to fulfill its molecular tasks (Fig. 7C). On the other side, *CD24* deficiency led to a decrease in ectodermal marker genes associated with neuronal, olfactory, and eye‐related differentiation (Fig. 7C). Furthermore, our mass spectrometric analysis suggested that CD24 is involved in modifying the chromatin and/or proteins by acetylation, methylation, ubiquitination, or citrullination. Thus, CD24 also utilizes/influences epigenetic mechanisms to mediate its functions. In conclusion, CD24 has a bivalent function in regulating developmental and differentiation processes by negatively and positively regulating glycoproteins; that is, it blocks the germ cell program and mesodermal differentiation and is able to poise cells for ectodermal differentiation. In line, *CD24*‐expressing neural precursor cells generated from pluripotent stem cells effectively differentiated along the neuronal lineage *in vitro* [95].

In our IHC analysis, CD24 was absent in germ cells of different developmental stages, GCNIS cells, and upregulated during reprogramming to an EC, which closely resemble pluripotent embryonal stem cells. Seminomas, which mimic PGC/GCNIS cells, were also CD24^‐^. Thus, we hypothesize that in GCT entities resembling (primordial) germ cells (GCNIS, seminoma), CD24 is absent, allowing for expression of germ cell factors, while in embryonal stem cell‐like ECs, which show the least relationship to germ cells, CD24 is involved in blocking the germ cell program.

Finally, we demonstrated that targeting CD24 might be reasonable approach to enhance efficacy of cisplatin to treat (cisplatin‐resistant) GCT cells. So far, several CD24 targeting strategies based on the SWA11 antibody exist, for example an antibody–drug conjugate (CD24‐PE38) and 3^rd^‐generation CD24‐CAR‐NK cells [96, 97]. Furthermore, the SWA11 antibody used in this study has already been shown to reduce tumorigenicity *in vivo* [51, 88]. Thus, in future studies the suitability of targeting CD24 in the treatment of (refractory) GCTs has to be evaluated in more detail.

## 5 Conclusion

In summary, CD24 is involved in suppressing the germ cell program during formation of ECs and promotes differentiation rather into ectodermal than mesodermal lineage upon differentiation‐inducing stimuli. *CD24* expression can be transactivated by the pluripotency factor SOX2 and reactivated in CD24^‐^ GCTs by histone deacetylase inhibitors or DNA‐demethylating agents. In conclusion, this study deciphered the molecular function CD24 in ECs and its (epi)genetic mechanisms of regulation, setting the stage for further analyses on the suitability of CD24 as a therapeutic target in ECs.

## Conflict of interest

The authors declare no conflict of interest.

## Author contributions

DN, HS, GK, and PAl conceptualized the study. MAS, TKB, HS, and DN performed data curation. MAS, SJ, FB, FF, TL, AS, PP, GK, HS, and DN performed formal analysis. MAS, TKB, LK, SJ, FB, FF, KF, MRM, AB, and GAW performed investigation. MAS, DN AS, KS, KK, HS, and DN contributed to methodology. DN, HS, DK, and Pal contributed to project administration. DN, HS, GK, and PAlt provided resources. MAS, PP, TL, KK, and KS contributed to software. DN, HS, and GK underwent supervision. MAS, TKB, LK, SJ, KF, MRM, and AB performed validation. DN, MAS, and FB performed visualization. DN wrote the original draft. DN, MAS, HS, GK, and PAl wrote, reviewed, and edited the manuscript.

## Ethics statement

The study methodologies conformed to the standards set by the Declaration of Helsinki. The study methodologies were approved by the local ethics committee. The ethics committee of the Heinrich Heine University Düsseldorf raised no concerns on using analyzed cell lines for *in vitro* experiments and drug screening (ethics votes 2018‐178 and 2019‐412 to DN). The ethics committees of the University Bonn and the University Medical Center Göttingen raised no concerns on performing analyses on GCT tissues of local biobanks. Experiments were undertaken with the understanding and written consent of each subject. All animal experiments were performed under license of the ‘Landesamt für Natur und Umwelt NRW’ (AZ‐84‐02.04.2013‐A430).

### 1 Data accessibility

RNA‐seq data are freely available via ‘Gene Expression Omnibus’ (GEO) (GSE168646). Illumina and Affymetrix expression microarray data are available via GEO (GSE71239, GSE71269, GSE79065, and GSE60698). The mass spectrometry proteomics data have been deposited to the ‘ProteomeXchange Consortium’ via the ‘PRIDE’ partner repository (PXD025110). Illumina Infinium MethylationEPIC bead array data are available via GEO (GSE176450).

## Supporting information


**Fig. S1**. (A) Illustration of *CD24* isoform expression in GCT and testis tissues as well as other cancer entities and corresponding tissues of origin based on the TCGA and GTEX cohorts. Location of ‘primers’ used in this study for qRT‐PCR analysis of various isoforms are indicated by purple arrow heads. (B) RNA sequencing data of *CD24* expression throughout various cancer types based on data extracted from TCGA. Data has been illustrated using ‘Firebrowse’. (C) Representative western blot analysis (n = 3) of CD24 protein levels (SWA11 antibody) in GCT cell lines and fibroblasts (MPAF). (D) Summary of immunohistochemistry data of indicated proteins in GCT tissues. (E) HE and immunohistochemical staining of CD24 in a mixed GCT composed of EC (OCT3/4^+^) and YST (FOXA2^+^) cells (picture of CD24 staining is also given in Fig. 1 C). (F) Immunohistochemical staining of CD24 in normal testis tissue. (G) Mutational burden of *CD24* in GCT tissues based on the TCGA ‘testicular germ cell tumor’ cohort. Data has been illustrated using ‘cBioPortal’.

**Fig. S2**. (A) CpG dinucleotide density and CpG island locations around the genomic region coding for *CD24*. Location of oligonucleotides used for sodium bisulfite sequencing analysis are indicated by purple arrow heads. Start of exon 1 is labeled by green arrow head. (B) Sodium bisulfite sequencing results of ten CpG dinucleotides in the *CD24* CpG island in GCT cell lines. Each cell line has been analyzed in sextuplicate. White circle: unmethylated CpG dinucleotide; black circle: methylated CpG dinucleotide. (C) Validation of CpG methylation data by methylation‐specific PCR of the region analyzed in (B) and the alternative promotor of the *CD24* main isoform (see Fig. 2 E, gray box). (D) Immunohistochemical staining of GCT tissues for CD24 (SWA11 antibody). The same samples as used for *CD24* CpG island DNA methylation analyses were stained. (E) Sodium bisulfite sequencing results (n = 6) of the *CD24* CpG island in JAR cells 96 h after application of 20 and 50 nM Decitabine.

**Fig. S3**. (A) XTT assay‐based analysis of viability over 96 h in GCT cells treated once with various concentrations of Quisinostat, JIB‐04, LP99, PRT4165 and GSK343. Each sample has been analyzed in quadruplicates. (B) Representative western blot analysis of histone H3 pan‐acetylation in Quisinostat‐treated (16 h, 5 nM) TCam‐2, 2102EP and JAR cells (n = 2). (C) Representative western blot analysis (n = 3) of CD24 (SWA11 antibody) in Quisinostat‐treated (16 h, 5 nM) 2102EP and JAR cells. (D) qRT‐PCR analysis of *CD24* expression (fold change to solvent control; n = 3) in 16 h Quisinostat‐treated JEG3 and BeWo cells (5 nM) as well as Entinostat‐treated TCam‐2 (2.45 µM), GCT72 (1.10 µM) and JAR (4.59 µM) cells. (E) qRT‐PCR analysis of *CD24* expression in TCam‐2 and JAR cells 48 ‐ 72 h after treatment with 50 or 100 nM recombinant GDF3 protein (n = 3). (F) Flow cytometry analysis of GFP‐ and mCherry‐positive GCT and control cells, respectively. (G) qRT‐PCR analysis (n = 3) of microenvironmental component marker genes in flow cytometry‐sorted GCT and control cell populations after coculture. Two‐tailed t‐tests were performed to test for significance; * = p‐value < 0.05, ** = p‐value < 0.005, *** = p‐value < 0.0005.

**Fig. S4**. (A) CRISPR/Cas9 and genotyping strategy to generate *CD24*‐deficient GCT cells and validate gene editing. In case of a successful gene editing by guide RNAs A, B and C, a 160 bp long fragment is amplified by PCR (PCR product 1). PCR product 2 represents a 234 bp ‘wild‐type’ band. (B) Agarose gel electrophoresis of wild‐type and NCCIT‐ / 2102EP‐Δ*CD24* clones demonstrated a successful gene editing. (C) Western blot analysis demonstrating absence of CD24 protein in NCCIT‐ / 2102EP‐Δ*CD24* cells. (D) Quality check of RNA used for RNA sequencing utilizing capillary electrophoresis (Fragment Analyzer). RQ values were calculated from band sizes / intensities. (E) Western blot analysis of CD24 protein levels and qRT‐PCR analysis of indicated marker genes 48h after *CD24* siRNA transfection in NCCIT cells. Scrambled RNA (scrRNA) served as negative control. (F) Representative densitometric analysis of DNA dot blot data (n = 3) using a 5mC antibody in three NCCIT / 2102EP‐Δ*CD24* clones and parental cells. Data were normalized against methylene blue staining (MB). Two‐tailed t‐tests were performed to test for significance; * = p‐value < 0.05, ** = p‐value < 0.005, *** = p‐value < 0.0005.

**Fig. S5**. (A) PCA of mass spectrometry data of NCCIT‐Δ*CD24* clones and parental cells. (B) Phyton‐based illustration of differentially regulated proteins in NCCIT‐Δ*CD24* cells compared to the parental cells. (C) DAVID‐based prediction of biological processes and molecular functions in which the proteins deregulated in NCCIT‐Δ*CD24* cells compared to the parental cells are involved in. (D) STRING‐based protein interaction prediction of the proteins upregulated or downregulated in NCCIT‐Δ*CD24* cells compared to the parental cells.

**Table S1**. Cell lines used in this study including cultivation conditions.

**Table S2**. Oligonucleotides used in this study.

**Table S3**. Antibodies used in this study.

**Table S4**. RNA seq, 850k DNA methylation array and MSA data.

## References

[1] Skakkebaek NE , Rajpert‐De Meyts E , Buck Louis GM , Toppari J , Andersson A‐M , Eisenberg ML , Jensen TK , Jørgensen N , Swan SH , Sapra KJ *et al*. (2015) Male reproductive disorders and fertility trends: influences of environment and genetic susceptibility. Physiol Rev 96, 55–97. 10.1152/physrev.00017.2015 PMC4698396 26582516

[2] Rajpert‐De Meyts E , McGlynn KA , Okamoto K , Jewett MAS & Bokemeyer C (2016) Testicular germ cell tumours. Lancet 387, 1762–1774. 26651223 10.1016/S0140-6736(15)00991-5

[3] Cheng L , Albers P , Berney DM , Feldman DR , Daugaard G , Gilligan T & Looijenga LHJ (2018) Testicular cancer. Nat Rev Dis Prim 4, 29. 30291251 10.1038/s41572-018-0029-0

[4] Oing C , Giannatempo P , Honecker F , Oechsle K , Bokemeyer C & Beyer J (2018) Palliative treatment of germ cell cancer. Cancer Treat Rev 71, 102–107. 30415106 10.1016/j.ctrv.2018.10.007

[5] Fang X , Zheng P , Tang J & Liu Y (2010) CD24: from A to Z. Cell Mol Immunol 7, 100–103. 20154703 10.1038/cmi.2009.119 PMC4001892

[6] Aigner S , Ruppert M , Hubbe M , Sammar M , Sthoeger Z , Butcher EC , Vestweber D , Altevogt P & Kaufmann SHE (1995) Heat stable antigen (mouse CD24) supports myeloid cell binding to endothelial and platelet P‐selectin. Int Immunol 7, 1557–1565. 8562500 10.1093/intimm/7.10.1557

[7] Majores M , Schindler A , Fuchs A , Stein J , Heukamp L , Altevogt P & Kristiansen G (2015) Membranous CD24 expression as detected by the monoclonal antibody SWA11 is a prognostic marker in non‐small cell lung cancer patients. BMC Clin Pathol 15, 19. 26578846 10.1186/s12907-015-0019-z PMC4647809

[8] Deng J , Gao G , Wang L , Wang T , Yu J & Zhao Z (2012) CD24 expression as a marker for predicting clinical outcome in human gliomas. J Biomed Biotechnol 2012, 517172. 22500096 10.1155/2012/517172 PMC3303885

[9] Zhang P , Zheng P & Liu Y (2019) Amplification of the CD24 gene is an independent predictor for poor prognosis of breast cancer. Front Genet 10, 560. 31244889 10.3389/fgene.2019.00560 PMC6581687

[10] Sun J , Feng D , Xi H , Luo J , Zhou Z , Liu Q , Chen Y & Shao Q (2020) CD24 blunts the sensitivity of retinoblastoma to vincristine by modulating autophagy. Mol Oncol 14, 1740–1759. 32394616 10.1002/1878-0261.12708 PMC7400807

[11] Wan X , Cheng C , Shao Q , Lin Z , Lu S & Chen Y (2016) CD24 promotes HCC progression via triggering Notch‐related EMT and modulation of tumor microenvironment. Tumor Biol 37, 6073–6084. 10.1007/s13277-015-4442-7 26608371

[12] Zhou Z , Li Y , Kuang M , Wang X , Jia Q , Cao J , Hu J , Wu S , Wang Z & Xiao J (2020) The CD24+ cell subset promotes invasion and metastasis in human osteosarcoma. Biomedicine (Taipei) 51, 102598. 10.1016/j.ebiom.2019.102598 PMC6948162 31901872

[13] Sung CO , Park W , Choi Y‐L , Ahn G , Song SY , Huh SJ , Bae DS , Kim BG & Lee JH (2010) Prognostic significance of CD24 protein expression in patients treated with adjuvant radiotherapy after radical hysterectomy for cervical squamous cell carcinoma. Radiother Oncol 95, 359–364. 20153907 10.1016/j.radonc.2010.01.007

[14] Zhang W , Yi B , Wang C , Chen D , Bae S , Wei S , Guo RJ , Lu C , Nguyen LL , Yang WH *et al*. (2016) Silencing of CD24 enhances the PRIMA‐1‐induced restoration of mutant p53 in prostate cancer cells. Clin Cancer Res 22, 2545–2554. 26712693 10.1158/1078-0432.CCR-15-1927 PMC4867243

[15] Farid RM , Sammour SAE , ElDin ZAS , Salman MI & Omran TI (2019) Expression of CD133 and CD24 and their different phenotypes in urinary bladder carcinoma. Cancer Manag Res 11, 4677–4690. 31213893 10.2147/CMAR.S198348 PMC6536712

[16] Ooki A , VandenBussche CJ , Kates M , Hahn NM , Matoso A , McConkey DJ , Bivalacqua TJ & Hoque MO (2018) CD24 regulates cancer stem cell (CSC)‐like traits and a panel of CSC‐related molecules serves as a non‐invasive urinary biomarker for the detection of bladder cancer. Br J Cancer 119, 961–970. 30327565 10.1038/s41416-018-0291-7 PMC6203855

[17] Wu X , Wang W , Lai X , Zhou Y , Zhou X , Li J , Liang Y , Zhu X , Ren X , Ding Y *et al*. (2020) CD24 and PRAME are novel grading and prognostic indicators for pineal parenchymal tumors of intermediate differentiation. Am J Surg Pathol 44, 11–20. 31567202 10.1097/PAS.0000000000001350

[18] Kristiansen G , Denkert C , Schlüns K , Dahl E , Pilarsky C & Hauptmann S (2002) CD24 is expressed in ovarian cancer and is a new independent prognostic marker of patient survival. Am J Pathol 161, 1215–1221. 12368195 10.1016/S0002-9440(10)64398-2 PMC1867310

[19] Gilad N , Zukerman H , Pick M & Gatt ME (2019) The role of CD24 in multiple myeloma tumorigenicity and effects of the microenvironment on its expression. Oncotarget 10, 5480–5491. 31534632 10.18632/oncotarget.27190 PMC6739209

[20] Alaterre E , Raimbault S & Goldschmidt H (2017) Prediction in patients with multiple myeloma. Oncotarget 8, 98931–98944. 29228738 10.18632/oncotarget.22131 PMC5716778

[21] Kristiansen G , Sammar M & Altevogt P (2004) Tumour biological aspects of CD24, a mucin‐like adhesion molecule. J Mol Histol 35, 255–262. 15339045 10.1023/b:hijo.0000032357.16261.c5

[22] Schönberger S , Kraft D , Nettersheim D , Schorle H , Casati A , Craveiro RB , Mohseni MM , Calaminus G & Dilloo D (2020) Targeting EpCAM by a bispecific trifunctional antibody exerts profound cytotoxic efficacy in germ cell tumor cell lines. Cancers (Basel) 12, 1279. 10.3390/cancers12051279 PMC7281168 32438548

[23] Siska PJ , Johnpulle RAN , Zhou A , Bordeaux J , Kim JY , Dabbas B , Dakappagari N , Rathmell JC , Rathmell WK , Morgans AK *et al*. (2017) Deep exploration of the immune infiltrate and outcome prediction in testicular cancer by quantitative multiplexed immunohistochemistry and gene expression profiling. Oncoimmunology 6, e1305535. 28507813 10.1080/2162402X.2017.1305535 PMC5414873

[24] Jostes S , Nettersheim D , Schneider S & Schorle H (2021) Standard cultivation of testicular germ cell cancer cell lines and establishment of CRISPR / Cas9‐knockout lines materials components for standard cell culture components for transfection of cell lines. Methods Mol Biol 2195, 85–97. 32852759 10.1007/978-1-0716-0860-9_7

[25] Skowron MA , Watolla MM & Nettersheim D (2021) Three‐dimensional cultivation of germ cell cancer cell lines as hanging drops. Methods Mol Biol 2195, 77–83. 32852758 10.1007/978-1-0716-0860-9_6

[26] Nettersheim D , Jostes S , Fabry M , Honecker F , Schumacher V , Kirfel J , Kristiansen G & Schorle H (2016) A signaling cascade including ARID1A, GADD45B and DUSP1 induces apoptosis and affects the cell cycle of germ cell cancers after romidepsin treatment. Oncotarget 7, 74931–74946. 27572311 10.18632/oncotarget.11647 PMC5342713

[27] Skowron MA , Vermeulen M , Winkelhausen A , Becker TK , Bremmer F , Petzsch P , Schönberger S , Calaminus G , Köhrer K , Albers P *et al*. (2020) CDK4 / 6 inhibition presents as a therapeutic option for paediatric and adult germ cell tumours and induces cell cycle arrest and apoptosis via canonical and non‐canonical mechanisms. Br J Cancer 123, 378–391. 32418994 10.1038/s41416-020-0891-x PMC7403155

[28] Pietschmann T , Heinkelein M , Heldmann M , Zentgraf H , Rethwilm A & Lindemann D (1999) Foamy virus capsids require the cognate envelope protein for particle export. J Virol 73, 2613–2621. 10074106 10.1128/jvi.73.4.2613-2621.1999 PMC104016

[29] Ou W , Marino MP , Suzuki A , Joshi B , Husain SR , Maisner A , Galanis E , Puri RK & Reiser J (2012) Specific targeting of human interleukin (IL)‐13 receptor α2‐positive cells with lentiviral vectors displaying IL‐13. Hum Gene Ther Methods 23, 137–147. 22612657 10.1089/hgtb.2012.054 PMC3848083

[30] Glaas MF , Wiek C , Wolter LM , Roellecke K , Balz V , Okpanyi V , Wagenmann M , Hoffmann TK , Grässlin R , Plettenberg C *et al*. (2018) Mutational and functional analysis of FANCB as a candidate gene for sporadic head and neck squamous cell Carcinomas. Anticancer Res 38, 1317–1325. 29491055 10.21873/anticanres.12354

[31] Genin M , Clement F , Fattaccioli A , Raes M & Michiels C (2015) M1 and M2 macrophages derived from THP‐1 cells differentially modulate the response of cancer cells to etoposide. BMC Cancer 15, 577. 26253167 10.1186/s12885-015-1546-9 PMC4545815

[32] Skowron MA , Hoffmann MJ , Watolla MM & Nettersheim D (2021) Evaluation of chemotherapeutic drugs for treatment of (cisplatin‐resistant) germ cell cancer cell lines. Methods Mol Biol 2195, 99–111. 32852760 10.1007/978-1-0716-0860-9_8

[33] Nettersheim D , Gillis A , Biermann K , Looijenga LHJ & Schorle H (2011) The seminoma cell line TCam‐2 is sensitive to HDAC inhibitor depsipeptide but tolerates various other chemotherapeutic drugs and loss of NANOG expression. Genes Chromosomes Cancer 50, 1033–1042. 21987446 10.1002/gcc.20918

[34] Nettersheim D , Biermann K , Gillis AJM , Steger K , Looijenga LHJ & Schorle H (2011) NANOG promoter methylation and expression correlation during normal and malignant human germ cell development. Epigenetics 6, 114–122. 20930529 10.4161/epi.6.1.13433 PMC3052918

[35] Du P , Zhang X , Huang C‐C , Jafari N , Kibbe WA , Hou L & Lin SM (2010) Comparison of Beta‐value and M‐value methods for quantifying methylation levels by microarray analysis. BMC Bioinformatics 11, 587. 21118553 10.1186/1471-2105-11-587 PMC3012676

[36] Nettersheim D , Jostes S , Sharma R , Schneider S , Hofmann A , Ferreira HJ , Hoffmann P , Kristiansen G , Esteller MB & Schorle H (2015) BMP inhibition in seminomas initiates acquisition of pluripotency via NODAL signaling resulting in reprogramming to an embryonal carcinoma. PLoS Genet 11, e1005415. 26226633 10.1371/journal.pgen.1005415 PMC4520454

[37] Nettersheim D , Berger D , Jostes S , Skowron M & Schorle H (2019) Deciphering the molecular effects of romidepsin on germ cell tumours: DHRS2 is involved in cell cycle arrest but not apoptosis or induction of romidepsin effectors. J Cell Mol Med 23, 670–679. 30460772 10.1111/jcmm.13971 PMC6307807

[38] Wruck W , Bremmer F , Kotthoff M , Fichtner A , Skowron MA , Schönberger S , Calaminus G , Vokuhl C , Pfister D , Heidenreich A *et al*. (2021) The pioneer and differentiation factor FOXA2 is a key driver of yolk‐sac tumour formation and a new biomarker for paediatric and adult yolk‐sac tumours. J Cell Mol Med 25, 1394–1405. 33448076 10.1111/jcmm.16222 PMC7875904

[39] Nettersheim D , Heukamp LC , Fronhoffs F , Grewe MJ , Haas N , Waha A , Honecker F , Waha A , Kristiansen G & Schorle H (2013) Analysis of TET expression/activity and 5mC oxidation during normal and malignant germ cell development. PLoS One 8, e82881. 24386123 10.1371/journal.pone.0082881 PMC3873252

[40] Jostes SV , Fellermeyer M , Arévalo L , Merges GE , Kristiansen G , Nettersheim D & Schorle H (2019) Unique and redundant roles of SOX2 and SOX17 in regulating the germ cell tumour fate. Int J Cancer 146, 1592–1605. 31583686 10.1002/ijc.32714

[41] Nettersheim D , Arndt I , Sharma R , Riesenberg S , Jostes S , Schneider S , Hölzel M , Kristiansen G & Schorle H (2016) The cancer/testis‐antigen PRAME supports the pluripotency network and represses somatic and germ cell differentiation programs in seminomas. Br J Cancer 115, 454–464. 27441500 10.1038/bjc.2016.187 PMC4985348

[42] Eckert D , Nettersheim D , Heukamp LC , Kitazawa S , Biermann K & Schorle H (2008) TCam‐2 but not JKT‐1 cells resemble seminoma in cell culture. Cell Tissue Res 331, 529–538. 18008088 10.1007/s00441-007-0527-y

[43] Jostes S , Nettersheim D , Fellermeyer M , Schneider S , Hafezi F , Honecker F , Schumacher V , Geyer M , Kristiansen G & Schorle H (2017) The bromodomain inhibitor JQ1 triggers growth arrest and apoptosis in testicular germ cell tumours in vitro and in vivo. J Cell Mol Med 21, 1300–1314. 28026145 10.1111/jcmm.13059 PMC5487916

[44] Nettersheim D , Heimsoeth A , Jostes S , Schneider S , Fellermeyer M , Hofmann A & Schorle H (2016) SOX2 is essential for in vivo reprogramming of seminoma‐like TCam‐2 cells to an embryonal carcinoma‐like fate. Oncotarget 7, 47095–47110. 27283990 10.18632/oncotarget.9903 PMC5216926

[45] Poschmann G , Seyfarth K , Besong Agbo D , Klafki H‐W , Rozman J , Wurst W , Wiltfang J , Meyer HE , Klingenspor M & Stühler K (2014) High‐fat diet induced isoform changes of the Parkinson’s disease protein DJ‐1. J Proteome Res 13, 2339–2351. 24646099 10.1021/pr401157k

[46] Tusher VG , Tibshirani R & Chu G (2001) Significance analysis of microarrays applied to the ionizing radiation response. Proc Natl Acad Sci USA 98, 5116–5121. 11309499 10.1073/pnas.091062498 PMC33173

[47] Perez‐Riverol Y , Csordas A , Bai J , Bernal‐Llinares M , Hewapathirana S , Kundu DJ , Inuganti A , Griss J , Mayer G , Eisenacher M *et al*. (2019) The PRIDE database and related tools and resources in 2019: improving support for quantification data. Nucleic Acids Res 47, D442–D450. 30395289 10.1093/nar/gky1106 PMC6323896

[48] Bremmer F , Bohnenberger H , Küffer S , Oellerich T , Serve H , Urlaub H , Strauss A , Maatoug Y , Behnes CL , Oing C *et al*. (2019) Proteomic comparison of malignant human germ cell tumor cell lines. Dis Markers 2019, 8298524. 31565104 10.1155/2019/8298524 PMC6745167

[49] Kurz L , Miklyaeva A , Skowron MA , Overbeck N , Poschmann G , Becker T , Eul K , Kurz T , Schönberger S , Calaminus G *et al*. (2020) ARID1A regulates transcription and the epigenetic landscape via POLE and DMAP1 while ARID1A deficiency or pharmacological inhibition sensitizes germ cell tumor cells to ATR inhibition. Cancers (Basel) 12, 905. 10.3390/cancers12040905 PMC7226530 32272809

[50] Kristiansen G , Machado E , Bretz N , Rupp C , Winzer K‐J , König A‐K , Moldenhauer G , Marmé F , Costa J & Altevogt P (2010) Molecular and clinical dissection of CD24 antibody specificity by a comprehensive comparative analysis. Lab Investig 90, 1102–1116. 20351695 10.1038/labinvest.2010.70

[51] Salnikov AV , Bretz NP , Perne C , Hazin J , Keller S , Fogel M , Herr I , Schlange T , Moldenhauer G & Altevogt P (2013) Antibody targeting of CD24 efficiently retards growth and influences cytokine milieu in experimental carcinomas. Br J Cancer 108, 1449–1459. 23511563 10.1038/bjc.2013.102 PMC3629417

[52] Nettersheim D , Jostes S & Schorle H (2017) Xenografting of cancer cell lines for in vivo screening of the therapeutic potential of HDAC inhibitors. Methods Mol Biol 1510, 211–215. 27761823 10.1007/978-1-4939-6527-4_15

[53] Shen H , Shih J , Hollern DP , Wang L , Bowlby R , Tickoo SK , Thorsson V , Mungall AJ , Newton Y , Hegde AM *et al*. (2018) Integrated molecular characterization of testicular germ cell tumors. Cell Rep 23, 3392–3406. 29898407 10.1016/j.celrep.2018.05.039 PMC6075738

[54] Cerami E , Gao J , Dogrusoz U , Gross BE , Sumer SO , Aksoy BA , Jacobsen A , Byrne CJ , Heuer ML , Larsson E *et al*. (2012) The cBio Cancer Genomics Portal: an open platform for exploring multidimensional cancer genomics data. Cancer Discov 2, 401–404. 22588877 10.1158/2159-8290.CD-12-0095 PMC3956037

[55] Gao J , Aksoy BA , Dogrusoz U , Dresdner G , Gross B , Sumer SO , Sun Y , Jacobsen A , Sinha R , Larsson E *et al*. (2013) Integrative analysis of complex cancer genomics and clinical profiles using the cBioPortal. Sci Signal 6, pl1. 23550210 10.1126/scisignal.2004088 PMC4160307

[56] Goldman M , Craft B , Brooks A , Zhu J & Haussler D (2020) Visualizing and interpreting cancer genomics data via the Xena platform. Nat Biotechnol 38, 675–678. 32444850 10.1038/s41587-020-0546-8 PMC7386072

[57] Du Y , Cai M , Xing X , Ji J , Yang E & Wu J (2021) PINA 3.0: mining cancer interactome. Nucleic Acids Res 49, D1351–D1357. 33231689 10.1093/nar/gkaa1075 PMC7779002

[58] Szklarczyk D , Gable AL , Lyon D , Junge A , Wyder S , Huerta‐Cepas J , Simonovic M , Doncheva NT , Morris JH , Bork P *et al*. (2019) STRING v11: Protein‐protein association networks with increased coverage, supporting functional discovery in genome‐wide experimental datasets. Nucleic Acids Res 47, D607–D613. 30476243 10.1093/nar/gky1131 PMC6323986

[59] Dennis G , Sherman BT , Hosack DA , Yang J , Gao W , Lane HC & Lempicki RA (2003) DAVID: database for annotation, visualization, and integrated discovery. Genome Biol 4, P3.12734009

[60] Hunter JD (2007) Matplotlib: A 2D graphics environment. Comput Sci Eng 9, 90–95.

[61] Waskom M (2021) Seaborn: statistical data visualization. J Open Source Softw 6, 3021.

[62] Metsalu T & Vilo J (2015) ClustVis: a web tool for visualizing clustering of multivariate data using Principal Component Analysis and heatmap. Nucleic Acids Res 43, W566–W570. 25969447 10.1093/nar/gkv468 PMC4489295

[63] Kubota H , Avarbock MR & Brinster RL (2003) Spermatogonial stem cells share some, but not all, phenotypic and functional characteristics with other stem cells. Proc Natl Acad Sci USA 100, 6487–6492. 12738887 10.1073/pnas.0631767100 PMC164473

[64] Li C , Heidt DG , Dalerba P , Burant CF , Zhang L , Adsay V , Wicha M , Clarke MF & Simeone DM (2007) Identification of pancreatic cancer stem cells. Cancer Res 67, 1030–1037. 17283135 10.1158/0008-5472.CAN-06-2030

[65] Zhu J , He J , Liu Y , Simeone DM & Lubman DM (2012) Identification of glycoprotein markers for pancreatic cancer CD24 +CD44 + stem‐like cells using nano‐LC‐MS/MS and tissue microarray. J Proteome Res 11, 2272–2281. 22335271 10.1021/pr201059g PMC3321127

[66] Liu H , Wang YJ , Bian L , Fang ZH , Zhang QY & Cheng JX (2016) CD44+/CD24+ cervical cancer cells resist radiotherapy and exhibit properties of cancer stem cells. Eur Rev Med Pharmacol Sci 20, 1745–1754. 27212166

[67] Zhang J , Chen X , Bian L , Wang Y & Liu H (2019) CD44 + /CD24 + ‐expressing cervical cancer cells and radioresistant cervical cancer cells exhibit cancer stem cell characteristics. Gynecol Obstet Invest 84, 174–182. 30317240 10.1159/000493129

[68] Wang M , Xiao J , Shen M , Yahong Y , Tian R , Zhu F , Jiang J , Du Z , Hu J , Liu W *et al*. (2011) Isolation and characterization of tumorigenic extrahepatic cholangiocarcinoma cells with stem cell‐like properties. Int J Cancer 128, 72–81. 20232394 10.1002/ijc.25317

[69] Yeung TM , Gandhi SC , Wilding JL , Muschel R & Bodmer WF (2010) Cancer stem cells from colorectal cancer‐derived cell lines. Proc Natl Acad Sci USA 107, 3722–3727. 20133591 10.1073/pnas.0915135107 PMC2840416

[70] Zhang C , Li C , He F , Cai Y & Yang H (2011) Identification of CD44+CD24+ gastric cancer stem cells. J Cancer Res Clin Oncol 137, 1679–1686. 21882047 10.1007/s00432-011-1038-5 PMC11828146

[71] Gao M , Bai H , Jethava Y , Wu Y , Zhu Y , Yang Y , Xia J , Cao H , Franqui‐Machin R , Nadiminti K *et al*. (2020) Identification and characterization of tumor‐initiating cells in multiple myeloma. J Natl Cancer Inst 112, 507–515. 31406992 10.1093/jnci/djz159 PMC7225664

[72] Wang R , Li Y , Tsung A , Huang H , Du Q , Yang M , Deng M , Xiong S , Wang X , Zhang L *et al*. (2018) INOS promotes CD24+CD133+ liver cancer stem cell phenotype through a TACE/ADAM17‐dependent Notch signaling pathway. Proc Natl Acad Sci USA 115, E10127–E10136. 30297396 10.1073/pnas.1722100115 PMC6205478

[73] Xiao W , Gao Z , Duan Y , Yuan W & Ke Y (2017) Notch signaling plays a crucial role in cancer stem‐like cells maintaining stemness and mediating chemotaxis in renal cell carcinoma. J Exp Clin Cancer Res 36, 41. 28279221 10.1186/s13046-017-0507-3 PMC5345133

[74] Leelawat K , Keeratichamroen S , Leelawat S & Tohtong R (2013) CD24 induces the invasion of cholangiocarcinoma cells by upregulating CXCR4 and increasing the phosphorylation of ERK1/2. Oncol Lett 6, 1439–1446. 24179538 10.3892/ol.2013.1587 PMC3813815

[75] Schabath H (2006) CD24 affects CXCR4 function in pre‐B lymphocytes and breast carcinoma cells. J Cell Sci 119, 314–325. 16390867 10.1242/jcs.02741

[76] Schech A , Kazi A , Yu S , Shah P & Sabnis G (2015) Histone deacetylase inhibitor entinostat inhibits tumor‐initiating cells in triple‐negative breast cancer cells. Mol Cancer Ther 14, 1848–1857. 26037781 10.1158/1535-7163.MCT-14-0778

[77] Kwon MJ , Han J , Seo JH , Song K , Jeong HM , Choi J‐S , Kim YJ , Lee S‐H , Choi Y‐L & Shin YK (2015) CD24 overexpression is associated with poor prognosis in luminal a and triple‐negative breast cancer. PLoS One 10, e0139112. 26444008 10.1371/journal.pone.0139112 PMC4596701

[78] Hüser L , Sachindra S , Granados K , Federico A , Larribère L , Novak D , Umansky V , Altevogt P & Utikal J (2018) SOX2‐mediated upregulation of CD24 promotes adaptive resistance toward targeted therapy in melanoma. Int J Cancer 143, 3131–3142. 29905375 10.1002/ijc.31609

[79] Ehira N , Oshiumi H , Matsumoto M , Kondo T , Asaka M & Seya T (2010) An embryo‐specific expressing TGF‐ family protein, growth‐differentiation factor 3 (GDF3), augments progression of B16 melanoma. J Exp Clin Cancer Res 29, 135. 20950440 10.1186/1756-9966-29-135 PMC2972255

[80] Aigner S , Ramos CL , Hafezi‐Moghadam A , Lawrence MB , Friederichs J , Altevogt P & Ley K (1998) CD24 mediates rolling of breast carcinoma cells on P‐selectin. FASEB J 12, 1241–1251. 9737727 10.1096/fasebj.12.12.1241

[81] Chen GY , Tang J , Zheng P & Liu Y (2009) CD24 and siglec‐10 selectively repress tissue damage – induced immune responses. Science 323, 1722–1725. 19264983 10.1126/science.1168988 PMC2765686

[82] Myung JH , Gajjar KA , Pearson RM , Launiere CA , Eddington DT & Hong S (2011) Direct measurements on CD24‐mediated rolling of human breast cancer MCF‐7 cells on E‐selectin. Anal Chem 83, 1078–1083. 21207944 10.1021/ac102901e PMC3059340

[83] Barkal AA , Brewer RE , Markovic M , Kowarsky M , Barkal SA , Zaro BW , Krishnan V , Hatakeyama J , Dorigo O , Barkal LJ *et al*. (2019) CD24 signalling through macrophage Siglec‐10 is a target for cancer immunotherapy. Nature 572, 392–396. 31367043 10.1038/s41586-019-1456-0 PMC6697206

[84] Hass R , von der Ohe J & Ungefroren H (2020) The intimate relationship among EMT, MET and TME: A t(ransdifferentiation) e(nhancing) M(ix) to be exploited for therapeutic purposes. Cancers (Basel) 12, 3674. 10.3390/cancers12123674 PMC7762343 33297508

[85] Ortiz‐Montero P , Liu‐Bordes WY , Londoño‐Vallejo A & Vernot JP (2018) CD24 expression and stem‐associated features define tumor cell heterogeneity and tumorigenic capacities in a model of carcinogenesis. Cancer Manag Res 10, 5767–5784. 30510447 10.2147/CMAR.S176654 PMC6248383

[86] Nakamura K , Terai Y , Tanabe A , Ono YJ , Hayashi M , Maeda K , Fujiwara S , Ashihara K , Nakamura M , Tanaka Y *et al*. (2017) CD24 expression is a marker for predicting clinical outcome and regulates the epithelial‐mesenchymal transition in ovarian cancer via both the Akt and ERK pathways. Oncol Rep 37, 3189–3200. 28440503 10.3892/or.2017.5583 PMC5442399

[87] Baumann P , Cremers N , Kroese F , Orend G , Chiquet‐Ehrismann R , Uede T , Yagita H & Sleeman JP (2005) CD24 expression causes the acquisition of multiple cellular properties associated with tumor growth and metastasis. Cancer Res 65, 10783–10793. 16322224 10.1158/0008-5472.CAN-05-0619

[88] Sagiv E , Starr A , Rozovski U , Khosravi R , Altevogt P , Wang T & Arber N (2008) Targeting CD24 for treatment of colorectal and pancreatic cancer by monoclonal antibodies or small interfering RNA. Cancer Res 68, 2803–2812. 18413748 10.1158/0008-5472.CAN-07-6463

[89] Vencken SF , Sethupathy P , Blackshields G , Spillane C , Elbaruni S , Sheils O , Gallagher MF & O’Leary JJ (2014) An integrated analysis of the SOX2 microRNA response program in human pluripotent and nullipotent stem cell lines. BMC Genom 15, 711. 10.1186/1471-2164-15-711 PMC4162954 25156079

[90] Caricasole AAD , van Schaik RHN , Zeinstra LM , Wierikx CDJ , van Gurp RJHLM , van den Pol M , Looijenga LHJ , Oosterhuis JW , Pera MF , Ward A *et al*. (1998) Human growth‐differentiation factor 3 (hGDF3): developmental regulation in human teratocarcinoma cell lines and expression in primary testicular germ cell tumours. Oncogene 16, 95–103. 9467948 10.1038/sj.onc.1201515

[91] de Jong J , Stoop H , Gillis AJM , van Gurp RJHLM , van de Geijn G‐JM , Boer M , Hersmus R , Saunders PTK , Anderson RA , Oosterhuis JW & *et al*. (2008) Differential expression of SOX17 and SOX2 in germ cells and stem cells has biological and clinical implications. J Pathol 215, 21–30. 18348160 10.1002/path.2332

[92] Muppala S , Mudduluru G , Leupold JH , Buergy D , Sleeman JP & Allgayer H (2013) CD24 induces expression of the oncomir miR‐21 via Src, and CD24 and Src are both post‐transcriptionally downregulated by the tumor suppressor miR‐34a. PLoS One 8, e59563. 23533633 10.1371/journal.pone.0059563 PMC3606220

[93] Ghuwalewala S , Ghatak D , Das S , Das P , Butti R , Gorain M , Kundu GC & Roychoudhury P (2019) MiR‐146a‐dependent regulation of CD24/AKT/β‐catenin axis drives cancer stem cell phenotype in oral squamous cell carcinoma. bioRxiv Prepr.

[94] Wang TW , Chern E , Hsu CW , Tseng KC & Chao HM (2020) SIRT1‐mediated expression of CD24 and epigenetic suppression of novel tumor suppressor MiR‐1185‐1 increases colorectal cancer stemness. Cancer Res 80, 5257–5269. 33046442 10.1158/0008-5472.CAN-19-3188

[95] Kim B‐J , Lee Y‐A , Kim K‐J , Kim Y‐H , Jung M‐S , Ha S‐J , Kang H‐G , Jung S‐E , Kim B‐G , Choi Y‐R *et al*. (2015) Effects of paracrine factors on CD24 expression and neural differentiation of male germline stem cells. Int J Mol Med 36, 255–262. 25976705 10.3892/ijmm.2015.2208

[96] Shapira S , Shapira A , Starr A , Kazanov D , Kraus S , Benhar I & Arber N (2011) An immunoconjugate of Anti‐CD24 and pseudomonas exotoxin selectively kills human colorectal tumors in mice. Gastroenterology 140, 935–946. 21147107 10.1053/j.gastro.2010.12.004

[97] Klapdor R , Wang S , Morgan M , Dörk T , Hacker U , Hillemanns P , Büning H & Schambach A (2019) Characterization of a novel third‐generation anti‐CD24‐CAR against ovarian cancer. Int J Mol Sci 20, 660. 10.3390/ijms20030660 PMC6387114 30717444

