# Supplementary figures and images for: The signal transducer CD24 suppresses the germ cell program and promotes an ectodermal rather than mesodermal cell fate in embryonal carcinomas
Source: Mol Oncol. 2021 Aug 2;16(4):982–1008. doi: 10.1002/1878-0261.13066 (PMC8847992; doi:10.1002/1878-0261.13066)

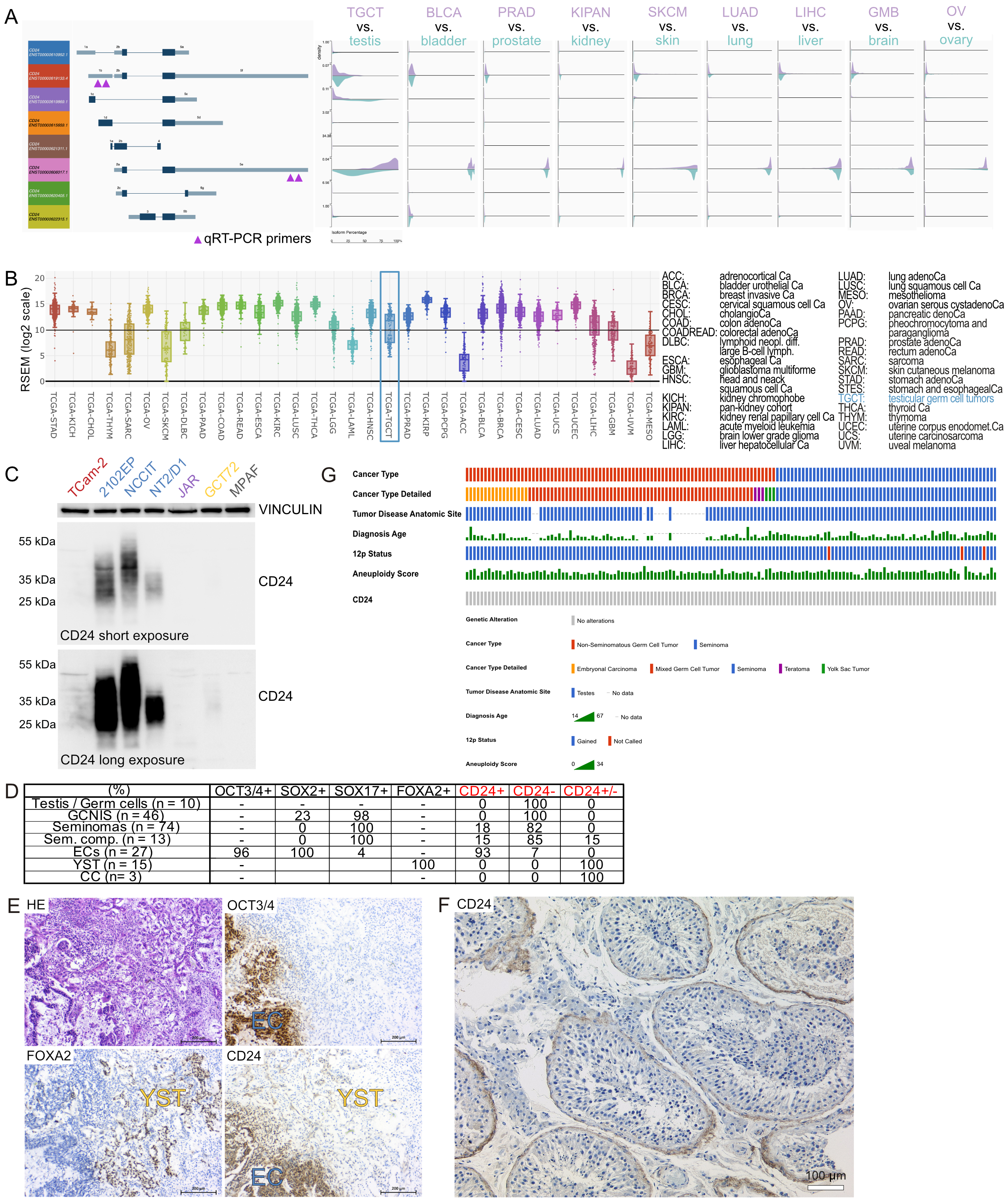

Supplement: Supplementary file 1 — Fig. S1. (A) Illustration of CD24 isoform expression in GCT and testis tissues as well as other cancer entities and corresponding tissues of origin based on the TCGA and GTEX cohorts. Location of ‘primers’ used in this study for qRT‐PCR analysis of various isoforms are indicated by purple arrow heads. (B) RNA sequencing data of CD24 expression throughout various cancer types based on data extracted from TCGA. Data has been illustrated using ‘Firebrowse’. (C) Representative western blot analysis (n = 3) of CD24 protein levels (SWA11 antibody) in GCT cell lines and fibroblasts (MPAF). (D) Summary of immunohistochemistry data of indicated proteins in GCT tissues. (E) HE and immunohistochemical staining of CD24 in a mixed GCT composed of EC (OCT3/4+) and YST (FOXA2+) cells (picture of CD24 staining is also given in Fig. 1 C). (F) Immunohistochemical staining of CD24 in normal testis tissue. (G) Mutational burden of CD24 in GCT tissues based on the TCGA ‘testicular germ cell tumor’ cohort. Data has been illustrated using ‘cBioPortal’. [file MOL2-16-982-s002.tiff]

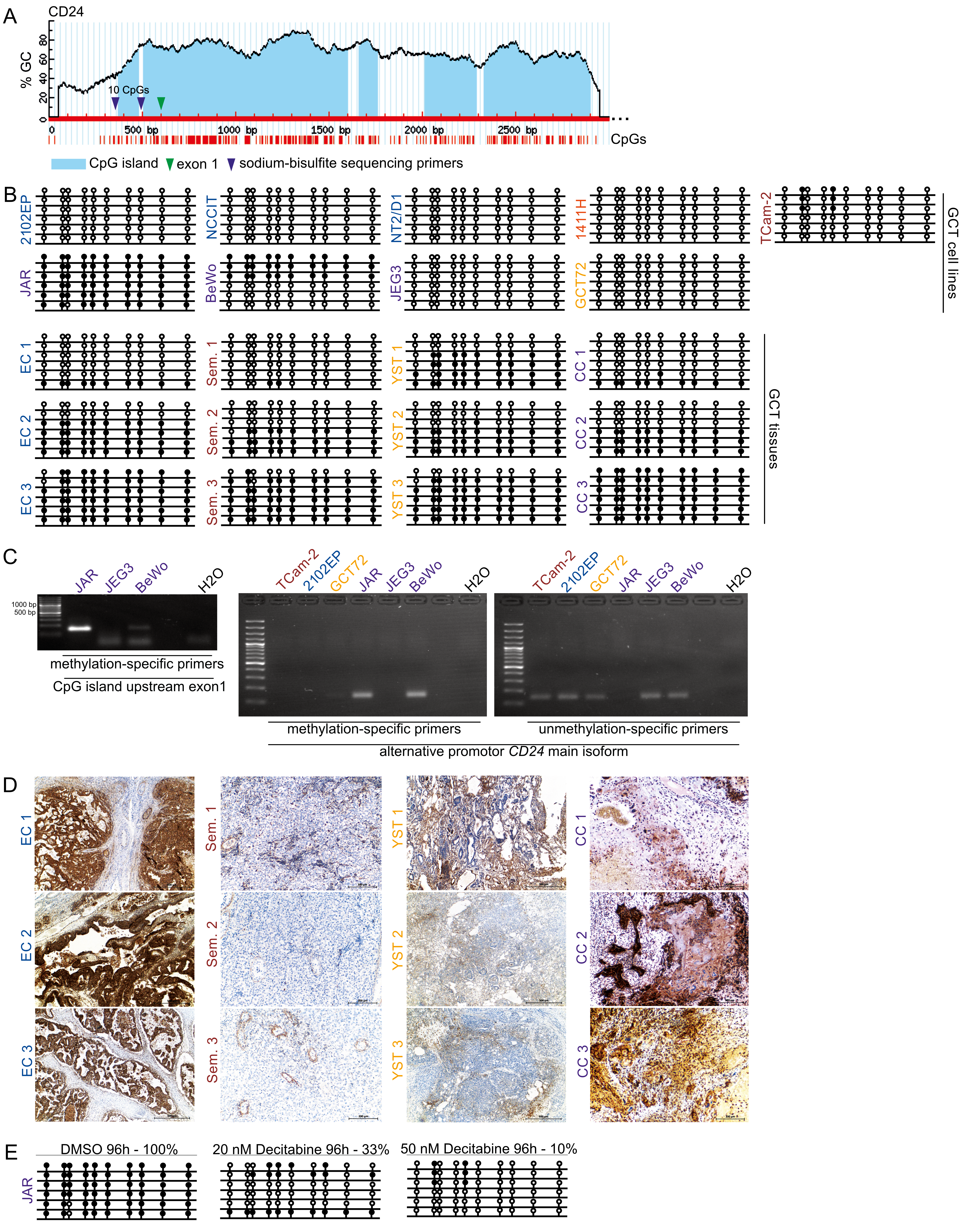

Supplement: Supplementary file 2 — Fig. S2. (A) CpG dinucleotide density and CpG island locations around the genomic region coding for CD24. Location of oligonucleotides used for sodium bisulfite sequencing analysis are indicated by purple arrow heads. Start of exon 1 is labeled by green arrow head. (B) Sodium bisulfite sequencing results of ten CpG dinucleotides in the CD24 CpG island in GCT cell lines. Each cell line has been analyzed in sextuplicate. White circle: unmethylated CpG dinucleotide; black circle: methylated CpG dinucleotide. (C) Validation of CpG methylation data by methylation‐specific PCR of the region analyzed in (B) and the alternative promotor of the CD24 main isoform (see Fig. 2 E, gray box). (D) Immunohistochemical staining of GCT tissues for CD24 (SWA11 antibody). The same samples as used for CD24 CpG island DNA methylation analyses were stained. (E) Sodium bisulfite sequencing results (n = 6) of the CD24 CpG island in JAR cells 96 h after application of 20 and 50 nM Decitabine. [file MOL2-16-982-s007.tiff]

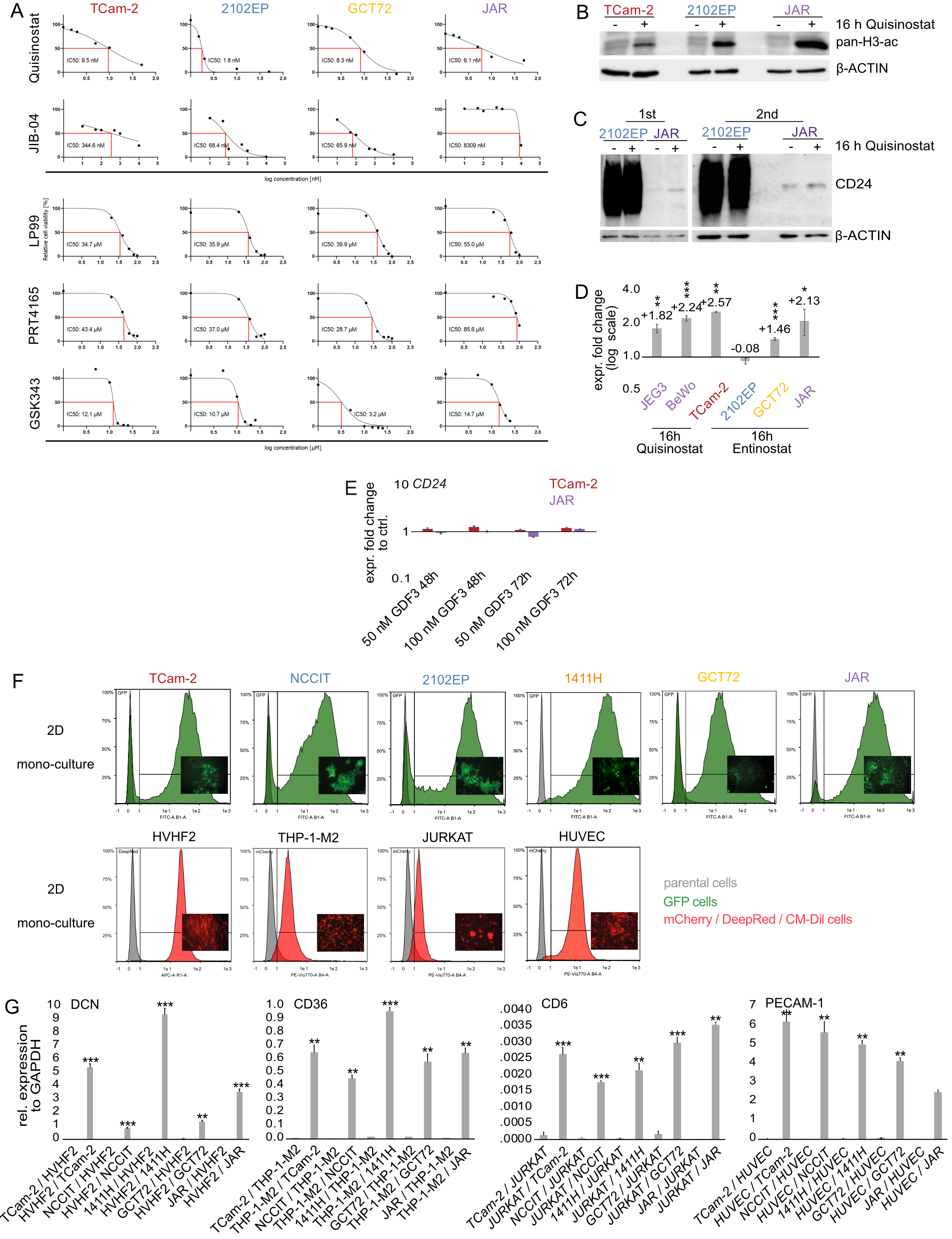

Supplement: Supplementary file 3 — Fig. S3. (A) XTT assay‐based analysis of viability over 96 h in GCT cells treated once with various concentrations of Quisinostat, JIB‐04, LP99, PRT4165 and GSK343. Each sample has been analyzed in quadruplicates. (B) Representative western blot analysis of histone H3 pan‐acetylation in Quisinostat‐treated (16 h, 5 nM) TCam‐2, 2102EP and JAR cells (n = 2). (C) Representative western blot analysis (n = 3) of CD24 (SWA11 antibody) in Quisinostat‐treated (16 h, 5 nM) 2102EP and JAR cells. (D) qRT‐PCR analysis of CD24 expression (fold change to solvent control; n = 3) in 16 h Quisinostat‐treated JEG3 and BeWo cells (5 nM) as well as Entinostat‐treated TCam‐2 (2.45 µM), GCT72 (1.10 µM) and JAR (4.59 µM) cells. (E) qRT‐PCR analysis of CD24 expression in TCam‐2 and JAR cells 48 ‐ 72 h after treatment with 50 or 100 nM recombinant GDF3 protein (n = 3). (F) Flow cytometry analysis of GFP‐ and mCherry‐positive GCT and control cells, respectively. (G) qRT‐PCR analysis (n = 3) of microenvironmental component marker genes in flow cytometry‐sorted GCT and control cell populations after coculture. Two‐tailed t‐tests were performed to test for significance; * = p‐value < 0.05, ** = p‐value < 0.005, *** = p‐value < 0.0005. [file MOL2-16-982-s008.tiff]

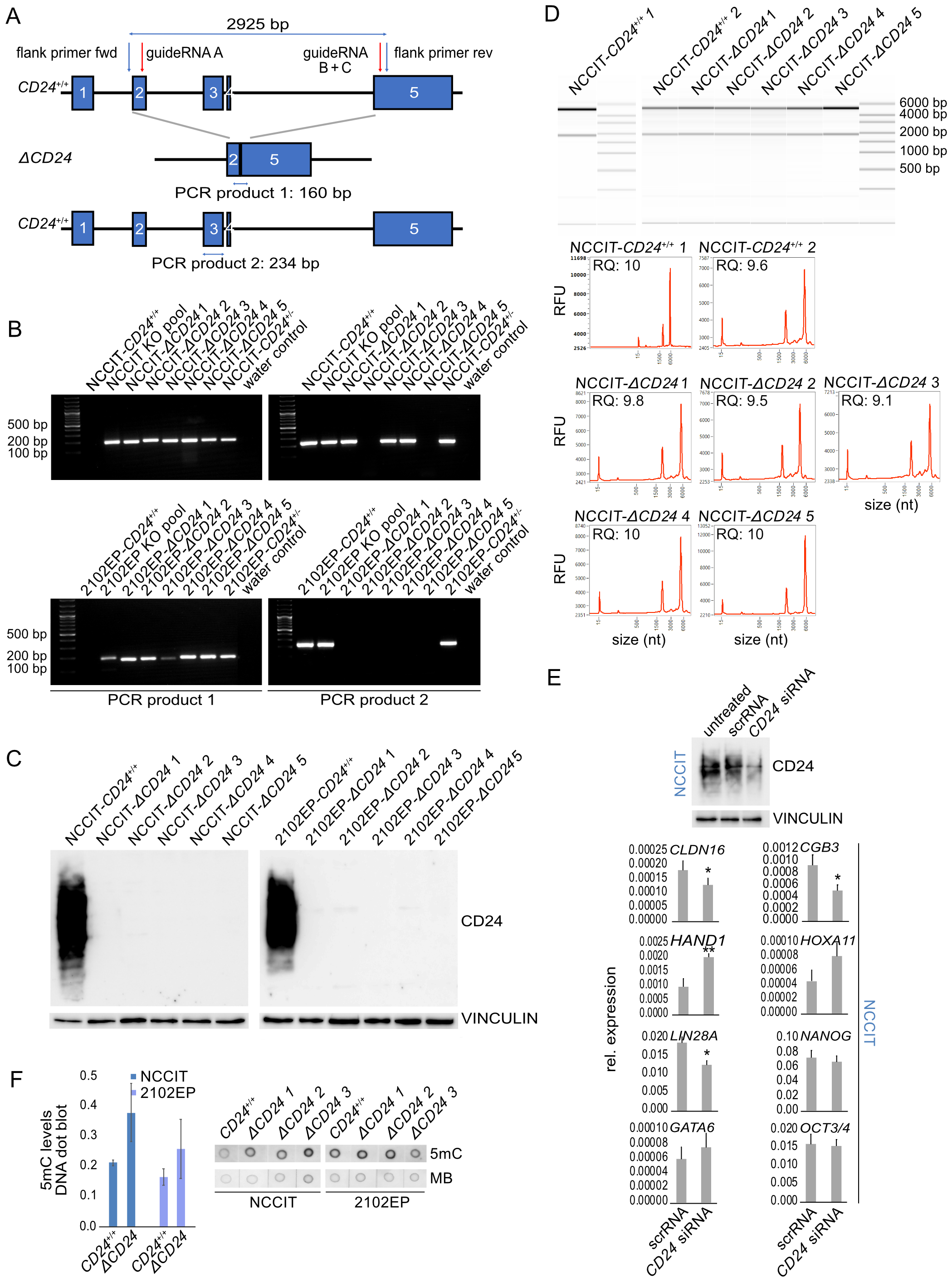

Supplement: Supplementary file 4 — Fig. S4. (A) CRISPR/Cas9 and genotyping strategy to generate CD24‐deficient GCT cells and validate gene editing. In case of a successful gene editing by guide RNAs A, B and C, a 160 bp long fragment is amplified by PCR (PCR product 1). PCR product 2 represents a 234 bp ‘wild‐type’ band. (B) Agarose gel electrophoresis of wild‐type and NCCIT‐ / 2102EP‐ΔCD24 clones demonstrated a successful gene editing. (C) Western blot analysis demonstrating absence of CD24 protein in NCCIT‐ / 2102EP‐ΔCD24 cells. (D) Quality check of RNA used for RNA sequencing utilizing capillary electrophoresis (Fragment Analyzer). RQ values were calculated from band sizes / intensities. (E) Western blot analysis of CD24 protein levels and qRT‐PCR analysis of indicated marker genes 48h after CD24 siRNA transfection in NCCIT cells. Scrambled RNA (scrRNA) served as negative control. (F) Representative densitometric analysis of DNA dot blot data (n = 3) using a 5mC antibody in three NCCIT / 2102EP‐ΔCD24 clones and parental cells. Data were normalized against methylene blue staining (MB). Two‐tailed t‐tests were performed to test for significance; * = p‐value < 0.05, ** = p‐value < 0.005, *** = p‐value < 0.0005. [file MOL2-16-982-s006.tiff]

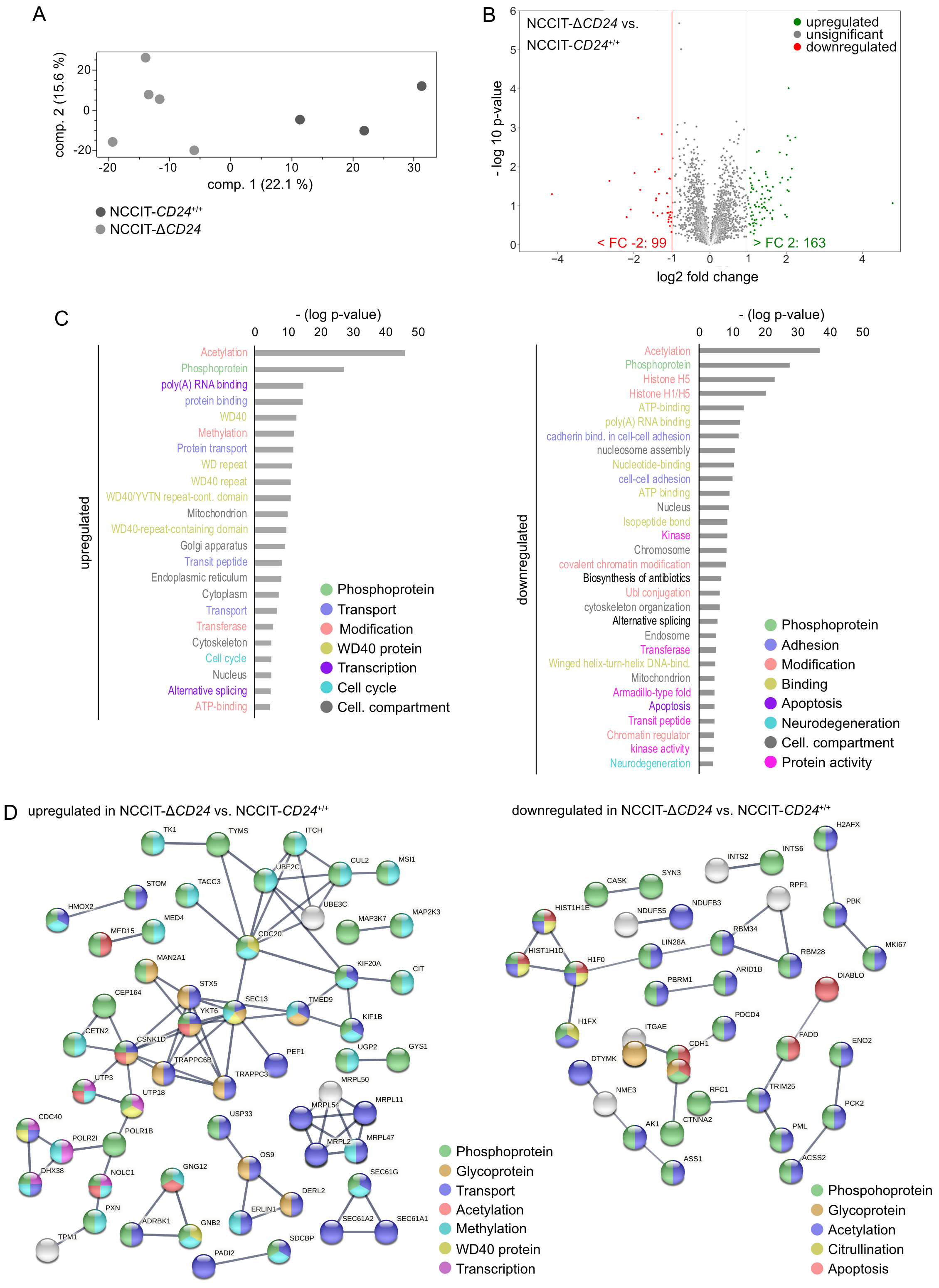

Supplement: Supplementary file 5 — Fig. S5. (A) PCA of mass spectrometry data of NCCIT‐ΔCD24 clones and parental cells. (B) Phyton‐based illustration of differentially regulated proteins in NCCIT‐ΔCD24 cells compared to the parental cells. (C) DAVID‐based prediction of biological processes and molecular functions in which the proteins deregulated in NCCIT‐ΔCD24 cells compared to the parental cells are involved in. (D) STRING‐based protein interaction prediction of the proteins upregulated or downregulated in NCCIT‐ΔCD24 cells compared to the parental cells. [file MOL2-16-982-s005.tiff]
